# When Appearances Deceive: Rape Myth Schemas Influence Attractiveness Effects Across Cultures

**DOI:** 10.1002/ijop.70256

**Published:** 2026-08-02

**Authors:** Ádám Putz, Zoë Marleen Cheryl Brinkert, Ayşe Naz Hazal Sezen, Amy E. Coren

**Affiliations:** ^1^ Department of Cognitive and Evolutionary Psychology University of Pécs Pécs Hungary; ^2^ Evolutionary and Cognitive Psychology Doctoral Program University of Pécs Pécs Hungary; ^3^ Department of Psychology Uskudar University Istanbul Türkiye; ^4^ Department of Psychology Pasadena City College Pasadena California USA

**Keywords:** attractiveness bias, culture, rape myth acceptance, sexual assault, victim blaming

## Abstract

Research on sexual assault judgements has shown that extra‐evidentiary cues influence perceptions of victims' credibility and responsibility. This study examined how physical attractiveness, rape myth acceptance (RMA), participant sex and cultural context shape victim blame judgements in samples from the United States, Hungary and Türkiye (*N* = 979). Participants evaluated sexual assault vignettes depicting different perpetrator‐victim attractiveness pairings and completed the Illinois Rape Myth Acceptance Scale. Victim blame was highest when an unattractive victim was paired with an attractive perpetrator. Turkish participants attributed greater blame to victims than did participants from the United States and Hungary, whereas the participants' sex had no overall effect. Controlling for RMA eliminated the attractiveness effect, suggesting that appearance‐based judgements are rooted in rape myth schemas rather than independent attractiveness biases. Mediation analyses showed that RMA explained the relationship between participant sex and victim blaming across nearly all conditions and accounted for the differences between the Hungarian and US samples. However, RMA did not explain the elevated victim blaming observed in the Turkish sample. These findings support a schema‐based interpretation of rape myths and suggest that cultural differences in victim blaming reflect additional sociocultural influences beyond rape myth endorsement.

## 1 Introduction

Victim blaming, attributing responsibility to survivors for assault, has significant consequences across the reporting‐to‐justice process. Higher blame increases stigma and reduces disclosure and reporting rates. It is linked to a lower conviction likelihood and lighter sentences, especially when juror‐like samples endorse rape myths and contributes to secondary victimisation (Hudspith et al. 2023). Thus, understanding when observers rely on attractiveness stereotypes and ideology to blame victims is crucial for both intervention and prevention efforts.

While the #MeToo movement has heightened awareness of sexual violence, reactions to allegations remain divided and shaped by ideology (White et al. 2025). Research on victim blaming has largely examined extra‐evidentiary characteristics—physical attractiveness, participant sex, rape myth acceptance (RMA) and cultural background—as independent predictors of judgement (Gravelin et al. 2019). Less is known about how these factors jointly influence evaluations of sexual assault allegations or why their effects vary across contexts. RMA—beliefs that deny or justify sexual aggression and shift responsibility from perpetrators to victims—is among the strongest predictors of victim blaming (Trottier et al. 2025), but it remains unclear whether it simply predicts blame or acts as a cognitive mechanism through which demographic and cultural influences operate. From a schema‐based perspective, rape myths may provide interpretive frameworks shaping how observers evaluate extra‐evidentiary information.

This perspective suggests that differences linked to participant sex, attractiveness, or cultural background may be partly explained by rape myth endorsement. The present study therefore investigated RMA's mediating role in a multicultural sample, examining how attractiveness, participant sex and cultural context influenced victim blame judgements and whether RMA explained individual and country‐level differences in judgements following sexual assault allegations.

### 1.1 Attractiveness Bias in Victim Blaming

Physical attractiveness influences evaluations of rape and sexual assault cases, although its effects depend on circumstances and whether the target is the victim or defendant. Physical attractiveness differs from other appearance‐based characteristics such as gender or ethnicity because it is a continuous evaluative dimension rather than membership in a social category. While categorical features activate stereotypes, attractiveness acts as a flexible heuristic cue producing evaluative biases across domains (Eagly et al. 1991). Accordingly, its influence depends on contextual interpretation and belief systems, such as rape myth acceptance, that shape appearance‐related judgements (Süssenbach et al. 2012).

Importantly, these inferences do not reflect an independent attractiveness‐based heuristic but are consistent with broader rape myth beliefs, including assumptions about who qualifies as a ‘real’ victim of sexual violence. Given the common assumption that sexual assault is motivated by sexual desire, observers may find allegations involving an attractive perpetrator and an unattractive victim less consistent with expectations of sexual assault (Gerdes et al. 1988; Zidenberg et al. 2021). Consequently, they may question the victim's account and assign greater responsibility to her (Vrij and Firmin 2001), inferring that she consented to or provoked the assault (Ferrão et al. 2016). Based on this reasoning, we predicted that unattractive victims would receive greater blame than attractive victims, particularly when paired with an attractive perpetrator (H1a).

Although rape myth acceptance (RMA) is a robust predictor of victim blaming, its interaction with attractiveness cues remains largely unexplored. Nason et al. (2021) found that men high in RMA judged women's responses to sexually coercive situations less favourably when the women were perceived as less attractive. Building on this finding, we hypothesise that attractiveness effects on victim blame will be attenuated or eliminated when RMA is statistically controlled (H1b), indicating that such effects reflect rape myth schemas rather than the independent influence of physical attractiveness.

### 1.2 Cultural Differences in Sexual‐Violence Perceptions

Recent evidence suggests that victim blaming may stem from multiple ideological pathways that vary across cultures (Milesi et al. 2020). Moral foundations related to authority, purity and social order influence victim blaming both directly and indirectly through modern myths about sexual aggression, but their relative importance differs across contexts. Consequently, cross‐cultural differences in victim blaming may reflect the varying influence of distinct belief systems rather than rape myth acceptance alone.

Culture shapes judgements of harm, credibility, and appropriate responses through norms concerning honour, dignity, gender and authority (Leung and Cohen 2011; Uskul et al. 2023). Honour cultures prioritise reputation and may tolerate aggression perceived as defending honour, whereas dignity cultures emphasise individual rights and institutional adjudication. Although national indicators such as the UNDP Gender Inequality Index (GII) capture gender‐related disparities, they should be viewed as proxies rather than direct measures of cultural beliefs. Existing cross‐cultural evidence suggests that victim blaming is higher in contexts characterised by stronger traditional gender ideologies (Pedersen and Strömwall 2013).

We compared the United States (often used as a dignity‐norm reference; GII = 0.195), Hungary (a post‐socialist EU member with mixed gender ideology indicators; GII = 0.242) and Türkiye (commonly examined in honour‐norm research; GII = 0.281).^1^ It is important to note that countries are heterogeneous, and we treat ‘nationality’ as a proxy for multiple sociocultural inputs (policy, media, religion, law, norms), rather than as essential cultural attributes. Drawing on these cultural logics, we expected victim blame to differ across countries (H2: Türkiye > Hungary > the United States). Such differences may reflect variation in baseline norms and in the application of attractiveness stereotypes and rape myth schemas.

### 1.3 Sex Differences in Victim Blaming and the Mediating Role of RMA

Victim blaming is often linked to observer characteristics such as gender. However, evidence for sex differences is inconsistent and appears to depend on contextual and stimulus characteristics (Gravelin et al. 2019; van der Bruggen and Grubb 2014). Moreover, increased awareness of sexual violence following #MeToo may have reduced the willingness to express victim‐blaming attitudes, particularly in self‐report measures (Szekeres et al. 2020). Consequently, apparent sex differences may be attenuated by social desirability concerns and shifting norms rather than genuine differences in underlying beliefs (PettyJohn et al. 2023; White et al. 2025). Given these developments and the greater explanatory value of ideological variables such as RMA, we predicted no main effect of participant sex on victim blame (H3).

Higher RMA is consistently associated with greater victim blame and reduced support for survivors (PettyJohn et al. 2023). Importantly, RMA functions as a cognitive schema that shapes attention, information processing and blame judgements in sexual assault cases (Süssenbach et al. 2012). Consistent with schema theory (Neisser, 1976, cited in Eyssel and Bohner 2011), RMA exerted a stronger influence on blame judgements when participants felt entitled to judge victims. Thus, demographic differences in victim blaming may reflect differences in rape myth endorsement rather than participant characteristics themselves. Accordingly, we hypothesised that RMA would mediate the relationship between participant sex and victim blame across all three national samples (H4a).

The same schema‐based logic can be extended to cultural differences. Because countries vary in normative environments, gender ideologies and cultural beliefs linked to rape myth acceptance (Pedersen and Strömwall 2013), cross‐national differences in victim blaming may partly reflect variation in RMA. Accordingly, we hypothesised that RMA would partially mediate the effect of nationality on victim blame judgements (H4b).

## 2 Method

The study employed a 2 (Perpetrator attractiveness: attractive vs. unattractive) × 2 (Victim attractiveness: attractive vs. unattractive) × 3 (Nationality: United States, Hungary, Türkiye) × 2 (Participant sex: male, female) mixed factorial design, with perpetrator and victim attractiveness as within‐subject factors and nationality and participant sex as between‐subject factors.

This study was conducted in accordance with the principles of the Declaration of Helsinki. This study was approved by the Hungarian United Ethical Review Committee for Research in Psychology (date: 3. December 2018/No. 2018‐106). As the data collection was conducted online, all participants provided informed consent electronically.

### 2.1 Sample

A total of 979 individuals participated in this study (Table 1). The US sample consisted of undergraduate psychology students from an urban US university, while the Hungarian and Turkish samples consisted of both university students and participants from the general population.^2^ Participation was voluntary and anonymous.

**TABLE 1:** Demographic data of the participants grouped by country and sex.

Nationality	Sex	*N*	Min.	Max.	*M*	SD
United States	Male	87	18	24	19.25	1.44
	Female	211	18	27	19.05	1.31
Hungary	Male	66	18	55	27.38	10.70
	Female	216	18	56	25.50	9.41
Türkiye	Male	169	18	61	35.86	11.06
	Female	230	18	59	33.04	10.52

Across all samples, 202 participants (21.6%) reported having experienced sexual assault at least once in their lifetime, of whom 85.6% were female. Because the victim blame scores were not normally distributed, Mann–Whitney U tests were conducted to compare participants with and without a history of sexual assault. The analyses revealed no significant differences in victim blame judgements across the attractiveness conditions (*p*'s > 0.05). Consequently, all subsequent analyses were conducted using the full sample to maximise sample heterogeneity and statistical power.

Because sample size was determined by participant availability, we conducted a post hoc sensitivity analysis based on the smallest hypothesis‐relevant effect: the victim attractiveness × nationality × participant sex interaction from the 2 × 2 × 3 × 2 mixed ANOVA (*η*_p_^2^ = 0.006). Using *α* = 0.05 and power = 0.80, approximately 1600 participants would have been required to detect an effect of this magnitude, whereas the final sample included 979 participants (61% of this threshold). Although the study was not optimally powered to detect such small higher‐order interactions, this represented the most demanding test in the analysis. The achieved sample size was sufficient for detecting the larger effects associated with the primary hypotheses, including the perpetrator attractiveness × victim attractiveness interaction and the main effect of nationality. Thus, the sensitivity analysis indicates adequate power for the principal analyses but more limited sensitivity for very small higher‐order effects.

### 2.2 Stimulus Materials and Manipulation Check

#### 2.2.1 Vignettes

**FIGURE 1:**
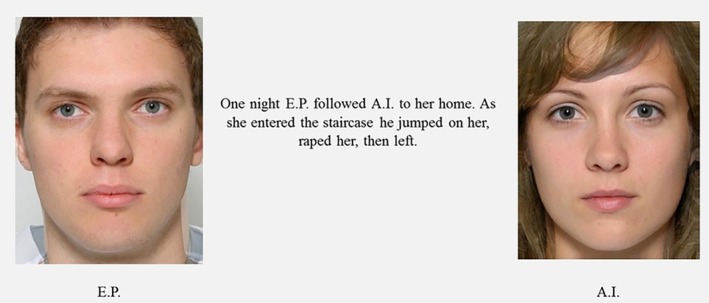
Example of vignettes used in this study. Male perpetrators always appeared on the left, while female victims appeared on the right, with sexual assault scenarios presented between the two faces.

For each national sample, eight vignettes were created, displaying both the male perpetrator and the female victim, accompanied by a brief description of the sexual assault scenario (Figure 1). Two vignettes represented each perpetrator–victim attractiveness pairing: attractive perpetrator/attractive victim, attractive perpetrator/unattractive victim, unattractive perpetrator/attractive victim and unattractive perpetrator/unattractive victim.

#### 2.2.2 Descriptions of Sexual Assault

Eight scenarios were selected from a pool of 12 sexual assault scenarios involving a male perpetrator and a female victim. The scenarios reflected the most common rape myths (e.g., the victim did not know the perpetrator, the offences occurred at night). Pilot studies were conducted in all three countries to standardise the amount of victim blame elicited by the scenarios. A series of one‐way ANOVAs indicated that the scenarios selected for inclusion in the study did not differ from each other in terms of the elicited amount of victim blame (*p'*s > 0.05).

#### 2.2.3 Perpetrator/Victim Photographic Stimuli

For the US and Hungarian samples, the photo stimuli of both the perpetrator and victim were selected from the authors' database of 89 previously synthesised images of Caucasian/White adults, both male and female, displaying neutral facial expressions (Putz et al. 2016). The same facial stimuli were used in both countries to maximise experimental comparability, and because the two samples were considered relatively similar in terms of their broader cultural and ethnic backgrounds, reducing the likelihood that the stimuli would be perceived differently across contexts. The photo stimuli were categorised into four groups based on earlier pilot study results: attractive males, attractive females, unattractive males and unattractive females. For privacy reasons, four individual portraits from the same category were selected and morphed together using PsychoMorph software, thus creating a total of eight attractive and eight unattractive averages for each gender as stimulus materials (Tiddeman et al. 2005).

A separate pilot study was conducted on a Turkish sample using 16 male and 16 female faces from the same database to create the visual stimuli for the Turkish participants. The study identified four attractive and four unattractive faces for each gender category. Unlike the US and Hungarian samples, the Turkish stimuli were based on individual photographs rather than morph composites, as the likelihood of participant recognition was considered negligible. All facial stimuli, pilot ratings and stimulus‐selection analyses are publicly available in the project's Open Science Framework (OSF) repository.^3^

#### 2.2.4 Manipulation Check

At the beginning of the study, before the experimental manipulation, participants were presented with photo stimuli, individually and in random order, using the PsyToolkit free online platform (Stoet 2010, 2017). For each photo, participants rated the face's physical attractiveness on a 7‐point Likert scale (1 = not at all attractive to 7 = extremely attractive). As the resulting data were not normally distributed, a series of Wilcoxon signed‐rank tests were applied to confirm that participants in all three samples rated the attractive stimulus material as significantly more attractive than the unattractive stimulus material (*p*'s < 0.001).

### 2.3 The Illinois Rape Myth Acceptance (IRMA) Scale

Participants' levels of rape myth acceptance were measured using the *Illinois Rape Myth Acceptance Scale*, which consists of 45 items with a 7‐point response scale (1 = not at all agree; 7 = very much agree) (Payne et al. 1999).^4^ The overall sum score was used as an indicator of the participants' level of rape myth acceptance (RMA). The internal consistency of the questionnaire was very high for all three samples; Cronbach's alpha values were 0.966, 0.949 and 0.923 for the United States, Hungarian and Turkish samples, respectively. The mean IRMA scores were 77.53 for the US sample, 94.60 for the Hungarian sample, and 76.09 for the Turkish sample.

### 2.4 Procedure

The study was conducted online using PsyToolkit. After providing informed consent, participants completed demographic questions and reported any history of sexual assault victimisation or perpetration; individuals under 18 were excluded. Participants then rated the attractiveness of the facial stimuli as a manipulation check before evaluating randomly presented vignettes and rating victim blame on a 7‐point scale (1 = Not at all to blame, 7 = Completely to blame). In the final phase, participants completed the IRMA scale. Because the study content could be distressing, contact information for the national sexual assault hotline was provided at the end of the survey. Completion time averaged 30 min across all three samples.

## 3 Results

A *p*‐value of less than 0.05 was considered statistically significant. Victim blame scores were averaged across the categories of stimuli: attractiveness of the perpetrator (attractive vs. unattractive) and attractiveness of the victim (attractive vs. unattractive), yielding four total victim blame scores for each participant.

### 3.1 The Influence of Perpetrator/Victim Attractiveness, Nationality and Participant's Sex on Victim Blaming

**FIGURE 2:**
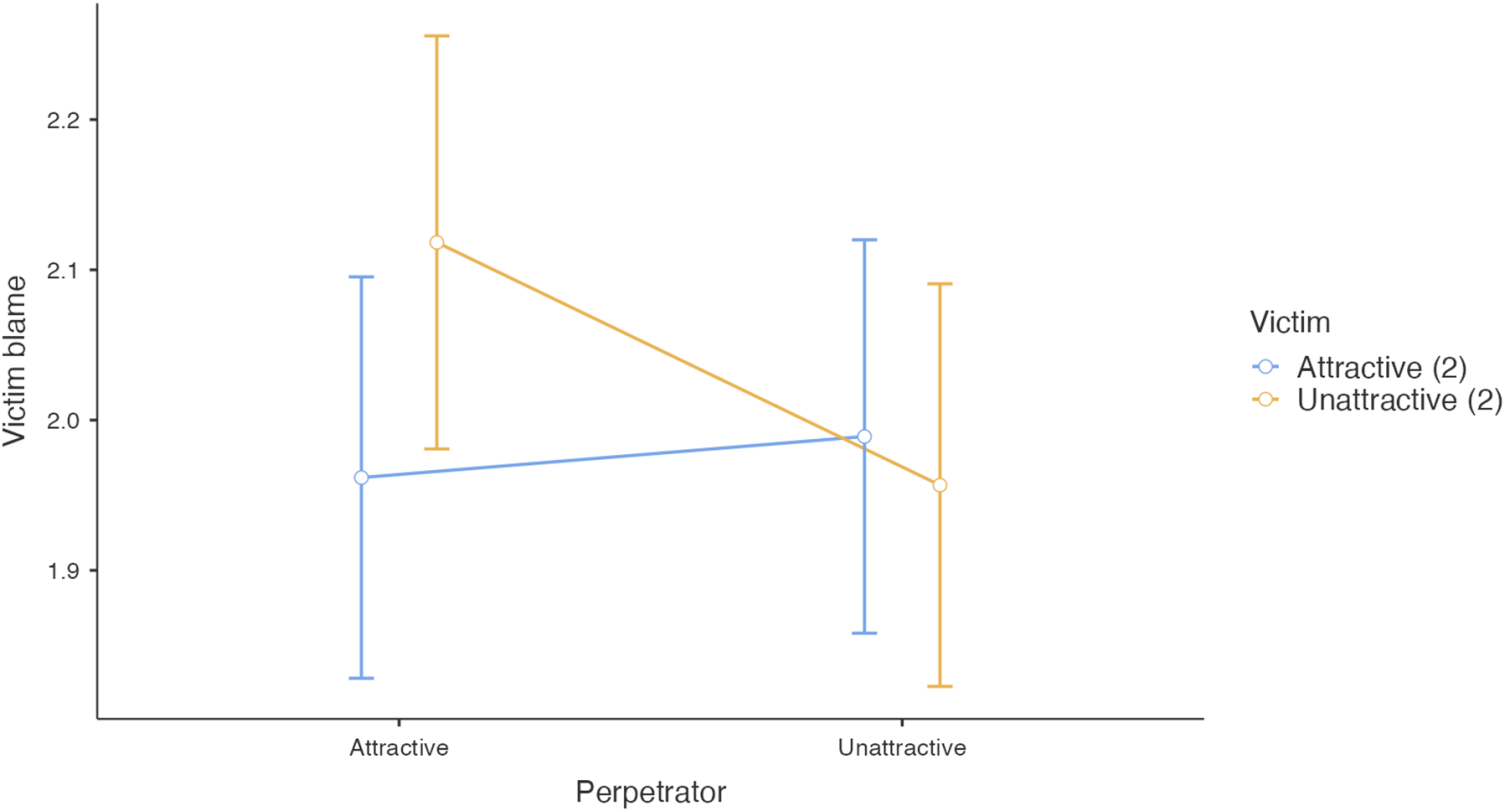
Mean victim blame scores as a function of perpetrator and victim attractiveness, illustrating the perpetrator attractiveness × victim attractiveness interaction. Error bars represent the 95% confidence intervals.

A 2 (Perpetrator: attractive, unattractive) × 2 (Victim: attractive, unattractive) × 3 (Nationality: United States, Hungarian, Turkish) × 2 (Participant sex: male, female) mixed ANOVA was conducted to examine the effects of physical attractiveness (perpetrator/victim), nationality and participant sex on participants' assessment of victim blame (H1‐3). Consistent with H1a, a significant perpetrator × victim interaction was observed, *F*(1, 973) = 31.41, *p* < 0.001, *η*_p_^2^ = 0.031. As shown in Figure 2, victim blame was highest when an unattractive victim was paired with an attractive perpetrator. Follow‐up pairwise comparisons using Tukey's HSD revealed that participants assigned significantly greater blame to unattractive than attractive victims when the perpetrator was attractive (*M* difference = −0.157, *t* = −5.41, *p* < 0.001). However, when the perpetrator was unattractive, victim attractiveness did not significantly influence blame judgements (*M* difference = 0.032, *t* = 1.46, *p* = 0.461). These findings suggest that the effect of victim attractiveness primarily emerged in cases involving attractive perpetrators.

In line with H1b, the perpetrator × victim interaction was no longer statistically significant in the ANCOVA analysis after controlling for participants' RMA scores, *F*(1, 972) = 2.16, *p* = 0.142. This pattern is consistent with the interpretation that individual differences in rape myth acceptance are associated with variance in victim blame judgements that overlap with attractiveness effects, suggesting that these effects may reflect the operation of rape myth schemas rather than an independent influence of physical attractiveness.

**FIGURE 3:**
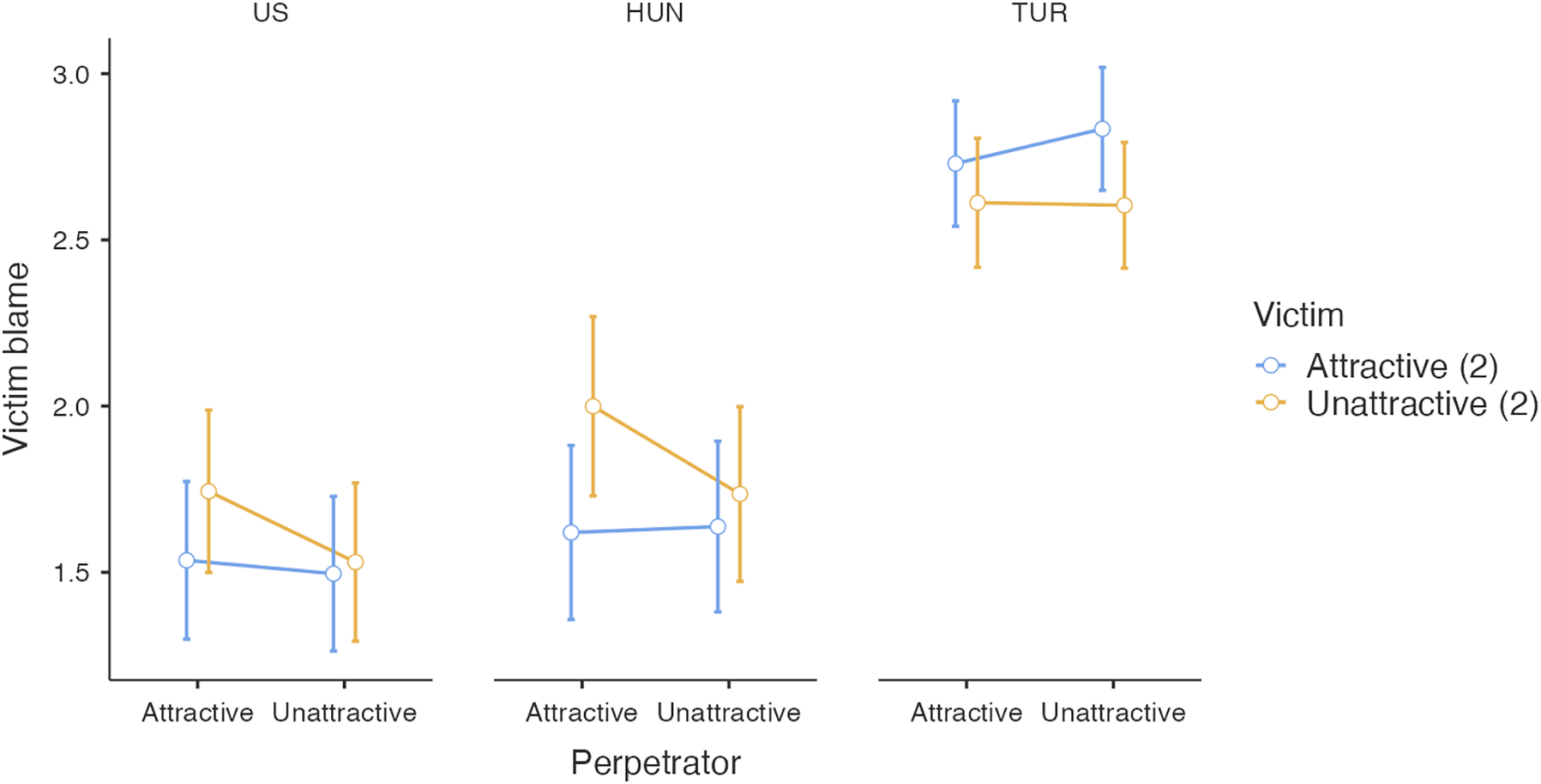
Mean victim blame scores as a function of perpetrator attractiveness, victim attractiveness, and nationality (the United States, Hungary and Türkiye). Error bars represent the 95% confidence intervals.

Consistent with H2, the analysis revealed a significant main effect of nationality, *F*(2, 973) = 33.47, *p* < 0.001, *η*_p_^2^ = 0.064. Turkish participants (*M* = 2.67, SD = 2.45) attributed significantly greater blame to victims than did participants from the United States (*M* = 1.54, SD = 1.34) and Hungary (*M* = 1.75, SD = 1.21). Contrary to our prediction, victim blame judgements did not differ significantly between the US and Hungarian samples. Although the perpetrator × victim × nationality interaction was not statistically significant, *F*(2, 973) = 2.18, *p* = 0.114, post hoc comparisons using Tukey's HSD revealed notable cross‐national differences in the pattern of blame attribution (Figure 3). Specifically, when the perpetrator was attractive, participants in both the United States (*M* difference = −0.208, *t* = −4.04, *p* = 0.003) and Hungary (*M* difference = −0.380, *t* = −6.69, *p* < 0.001) assigned significantly greater blame to unattractive victims than to attractive ones. However, this effect was substantially stronger in the Hungarian sample, where the magnitude of the difference was nearly twice that observed in the United States. A different pattern emerged in the Turkish sample, however. When the perpetrator was attractive, victim attractiveness did not significantly influence blame judgement (*M* difference = 0.118, *t* = 2.88, *p* = 0.148). In contrast, when the perpetrator was unattractive, Turkish participants attributed significantly greater blame to attractive than unattractive victims (*M* difference = 0.230, *t* = 7.35, *p* < 0.001). Together, these findings suggest that the relationship between attractiveness and victim blaming varies across cultural contexts, with the Turkish sample exhibiting a pattern that differed markedly from those observed in the US and Hungarian samples.

**FIGURE 4:**
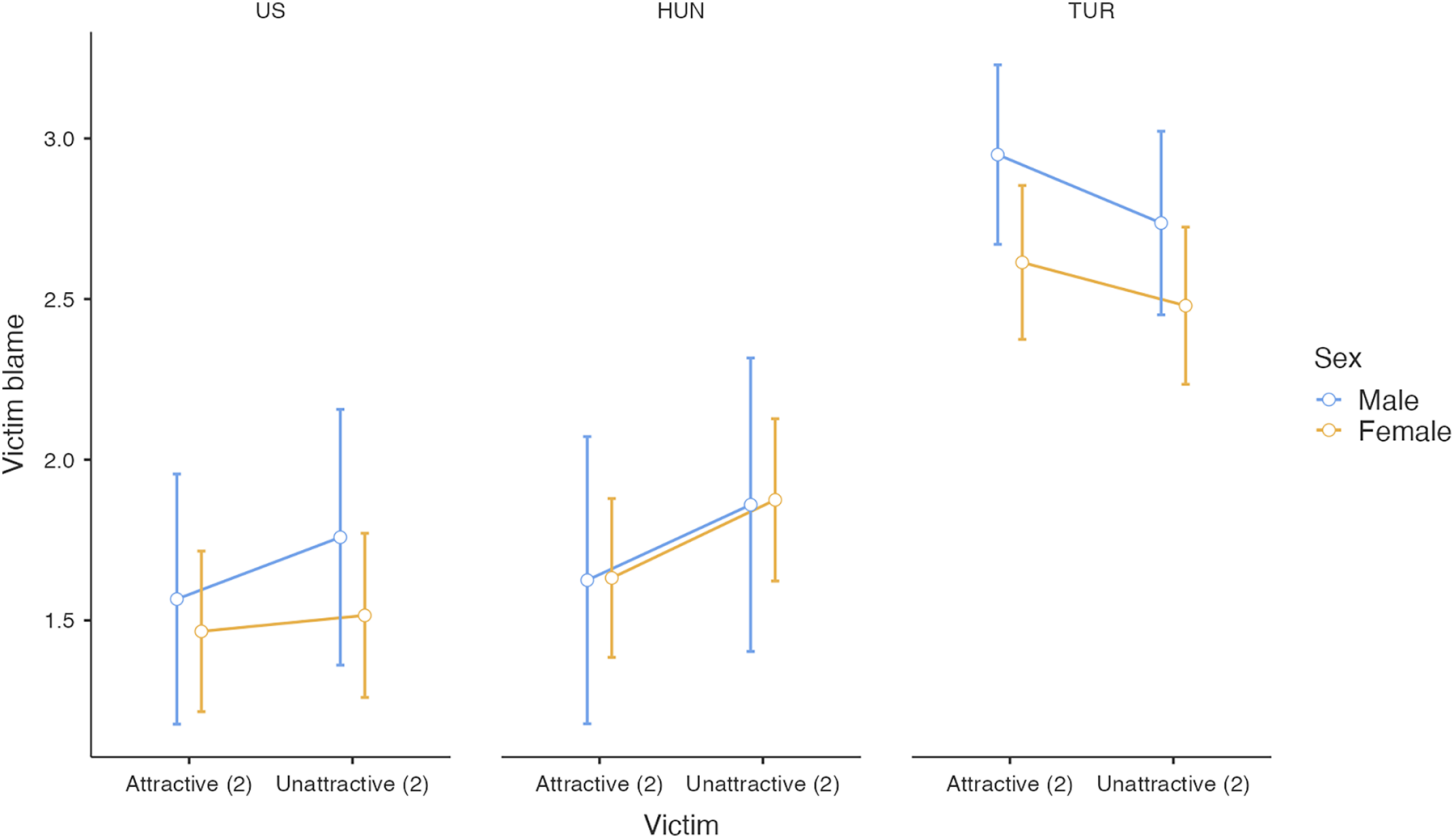
Mean victim blame scores as a function of victim attractiveness, participant sex and nationality (United States, Hungary and Türkiye). Error bars represent the 95% confidence intervals.

In line with H3, no overall sex difference in victim blame judgement was observed, *F*(1, 973) = 1.32, *p* = 0.251. However, a significant victim × nationality × participant sex interaction emerged, *F*(2, 973) = 3.13, *p* = 0.044, *η*_p_^2^ = 0.006, indicating that the relationship between victim attractiveness and blame attribution varied across countries and participant sex (Figure 4). Post hoc comparisons using Tukey's HSD revealed that in the United States, male participants attributed significantly greater blame to unattractive victims than to attractive ones (*M* difference = −0.193, *t* = −3.30, *p* = 0.046). In contrast, female participants did not differ in the amount of blame assigned to attractive versus unattractive victims (*M* difference = −0.050, *t* = −1.33, *p* = 0.975). In the Hungarian sample, both male (*M* difference = −0.193, *t* = −3.30, *p* = 0.046) and female participants (*M* difference = −0.193, *t* = −3.30, *p* = 0.046) assigned significantly greater blame to unattractive than to attractive victims. In contrast, the Turkish sample exhibited the opposite pattern. Both male (*M* difference = 0.213, *t* = 5.09, *p* < 0.001) and female participants (*M* difference = 0.135, *t* = 3.76, *p* = 0.010) attributed greater blame to attractive than unattractive victims.

Taken together, these findings suggest that the influence of victim attractiveness on blame judgements differs across cultural contexts and, to a lesser extent, by participant sex. Whereas attractiveness effects were primarily observed among men in the US sample, they were evident among both men and women in the Hungarian and Turkish samples, although the direction of the effect in Türkiye was reversed.

### 3.2 The Mediating Role of RMA in Victim Blaming

Mediation analyses were conducted to examine whether rape myth acceptance (RMA) mediated the relationship between participant sex and victim blame judgements across attractiveness conditions and national samples (H4a). Separate models were estimated for each combination of perpetrator and victim attractiveness within each country, resulting in 12 mediation analyses (see Supporting Information). Indirect effects were tested using bootstrapping procedures with 5000 resamples.

**FIGURE 5:**
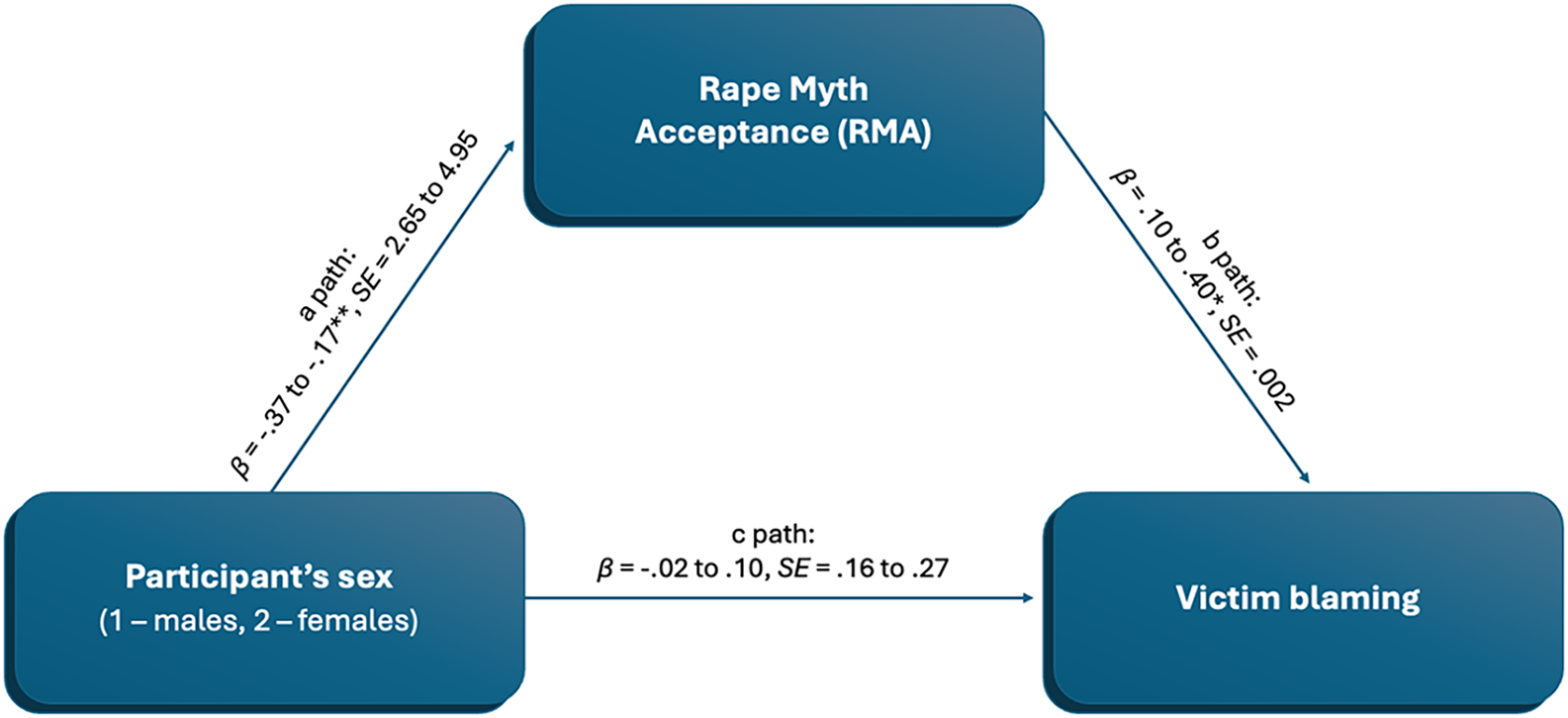
Aggregate results of the mediation analyses testing H4a. The figure summarises the indirect effect of participant sex on victim blame judgements through rape myth acceptance (RMA) across all national samples and perpetrator–victim attractiveness conditions. Path coefficients represent the range of standardised regression coefficients (β) and standard errors (SE) observed across the 12 mediation models. *p < 0.05, **p < 0.01.

Across all models, the direct effect of participant sex on victim blame, after controlling for the RMA score, was not statistically significant (*β*'s = −0.02 to 0.10, SE's = 0.16–0.27, *p*'s = 0.085–0.768). In contrast, the indirect effect of participant sex on victim blame through RMA was significant in 11 of the 12 models (*β*'s = −0.12 to −0.03, SE's = 0.06–0.09, *p*'s < 0.001–0.058). The only exception was the Turkish condition involving an attractive perpetrator and an unattractive victim, in which the indirect effect was only marginally significant. Examination of the individual mediation pathways indicated that participant sex significantly predicted RMA scores across all national samples (*β*'s = −0.37 to −0.17, *p*'s < 0.001–0.003), with men reporting higher levels of rape myth acceptance than women. RMA significantly predicted victim blame judgements across all attractiveness conditions (*β*'s = 0.10–0.40, *p*'*s* < 0.001–0.048), indicating that greater endorsement of rape myths was associated with increased victim blaming (Figure 5).

Taken together, these findings support the hypothesis that RMA mediates the relationship between participant sex and victim blame judgement (H4a). Although participant sex did not directly predict victim blaming, men tended to endorse rape myths to a greater extent than women, and these elevated levels of RMA were associated with greater blame attribution towards the victims.

To evaluate H4b, we conducted a series of mediation analyses to determine whether rape myth acceptance (RMA) accounted for cross‐national differences in victim blame judgements. Nationality was specified as a categorical predictor, with the US sample serving as the reference group, thereby allowing comparisons between Hungary and the United States and Türkiye and the United States. Separate models were estimated for each perpetrator–victim attractiveness combination, yielding four mediation analyses (see Supporting Information). Indirect effects were assessed using bias‐corrected bootstrapping with 5000 resamples.

**FIGURE 6:**
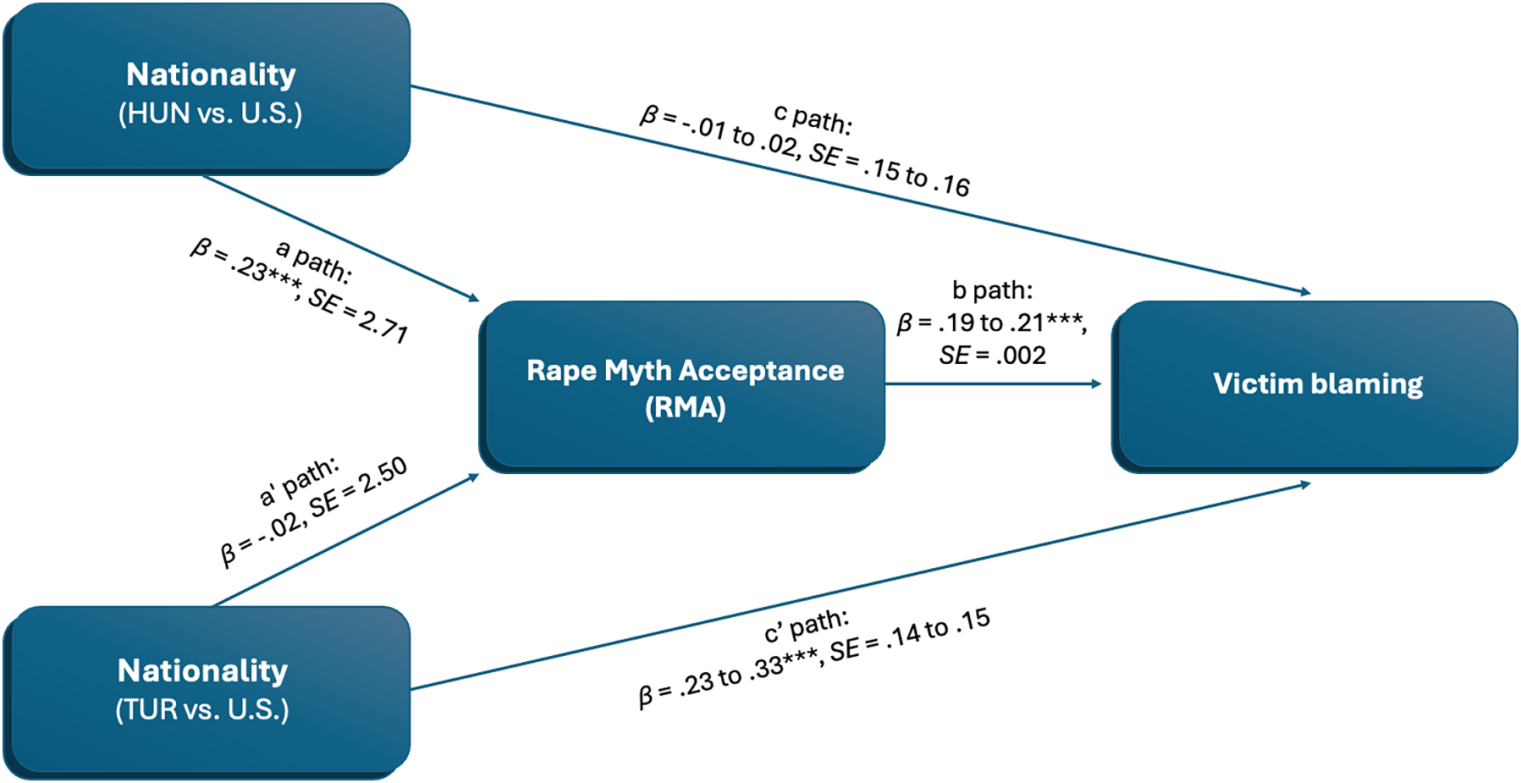
Aggregate results of the mediation analyses testing H4b. The figure summarises the relationships among nationality, rape myth acceptance (RMA) and victim blame judgements across the four perpetrator–victim attractiveness conditions. Nationality was coded using two contrasts (Hungary vs. the United States and Türkiye vs. the United States). Path coefficients represent the range of standardised regression coefficients (*β*) and standard errors (SE) observed across the mediation models. ***p < 0.001.

The mediation analyses revealed a consistent pattern across all four attractiveness conditions when comparing Hungarian and US participants. Nationality did not exert a significant direct effect on victim blame judgement, with standardised coefficients ranging from *β* = −0.01 to 0.02, SE's = 0.15–0.16 and *p*'s = 0.576–0.738. The corresponding 95% confidence intervals encompassed zero, indicating no direct relationship between nationality and victim blaming. However, the indirect effect of nationality (Hungary vs. the United States) on victim blame through rape myth acceptance (RMA) was significant in all four conditions, with *β*'s ranging from 0.04 to 0.05, SE's = 0.04–0.05, and *p*'s < 0.001. Further examination of the mediation pathways showed that nationality significantly predicted RMA scores (*β* = 0.23, SE = 2.71, *p* < 0.001), indicating that Hungarian participants reported higher levels of rape myth acceptance than their US counterparts. In turn, RMA emerged as a significant predictor of victim blame judgements across all conditions, with standardised coefficients ranging from *β* = 0.19 to 0.21 (*p*'s < 0.001). Taken together, these findings support an indirect pathway whereby Hungarian participants attributed greater blame to victims because they endorsed rape myths to a greater extent than US participants (Figure 6).

In contrast, comparisons between the Turkish and US samples revealed no significant indirect effect of nationality on victim blame through RMA across any of the mediation models. Indirect effects were negligible (*β* = 0.00, SE = 0.03, *p* = 0.566), and the 95% confidence intervals included zero, indicating that differences in rape myth acceptance did not account for the cross‐national variation in victim blaming between these groups. Instead, nationality exerted a significant direct effect on victim blame judgements, with standardised coefficients ranging from *β* = 0.23 to 0.33 (SE's = 0.14–0.15, *p*'s < 0.001). The corresponding 95% confidence intervals ranged from [0.63, 1.05] to [1.22, 1.61], indicating that Turkish participants consistently attributed greater blame to victims than US participants, irrespective of their levels of rape myth acceptance. These findings suggest that cultural differences between Türkiye and the United States influence victim blame judgements through mechanisms other than RMA. This supports the view that when comparing honour versus dignity cultures, broader sociocultural factors contribute to cross‐national variations in responses to sexual assault cases.

To examine whether demographic differences between the national samples influenced these findings, all mediation models were re‐estimated with participant age entered as a covariate (see Supporting Information). The inclusion of age did not materially alter the results. RMA continued to mediate the Hungary–United States differences in victim blaming, whereas the direct effect of nationality remained significant for Türkiye–United States comparisons. Thus, the observed mediation effects appear to be robust to age‐related variations across national samples.

## 4 Discussion

Consistent with H1a, participants attributed the greatest blame to unattractive victims paired with attractive perpetrators, supporting previous findings that attractiveness shapes judgements in sexual assault cases (Vrij and Firmin 2001). Such pairings may violate stereotypical expectations about sexual assault, increasing reliance on blame‐supportive interpretations (Ferrão et al. 2016). Consistent with H1b, this effect disappeared after controlling for RMA, suggesting that attractiveness influences blame through rape myth schemas rather than as an independent bias (Eyssel and Bohner 2011; Süssenbach et al. 2012).

The findings provided partial support for H2. Although Turkish participants consistently attributed greater blame to victims than participants from the United States and Hungary, no difference emerged between the latter two groups. This pattern supports the view that victim blaming may arise through multiple ideological pathways rather than broad cultural categories alone (Milesi et al. 2020). Notably, participants in the United States and Hungary blamed unattractive victims more, whereas Turkish participants sometimes blamed attractive victims more. While traditional accounts emphasise the credibility disadvantage of unattractive victims (Gravelin et al. 2019), this pattern is consistent with evidence that attractive victims may sometimes be viewed as more responsible for precipitating assault (Calhoun et al. 1978). Cultural beliefs concerning sexual availability, provocation, or respectability may help explain this reversal, although these constructs were not directly measured.

Consistent with H3 and H4a, participant sex had no direct effect on victim blame, but men reported higher RMA than women, and these differences explained sex‐related variation in victim blaming. This finding suggests that apparent demographic differences in blame judgements are better understood as differences in underlying belief systems. It also supports theoretical accounts of rape myths as cognitive frameworks that shape how individuals interpret and evaluate sexual assault allegations (Bohner et al. 2009; Eyssel and Bohner 2011; Süssenbach et al. 2012).

Support for H4b was also partial. RMA explained differences between the Hungarian and US samples but not the higher victim blaming observed in the Turkish sample. These findings suggest that rape myths explain some, but not all, cross‐national variation in victim blaming, consistent with evidence that culturally specific belief systems, including honour‐related norms, may shape judgements independently of rape myth endorsement (Gul and Schuster 2020; Milesi et al. 2020).

### 4.1 Practical Implications

Victim‐blaming judgements are shaped by rape myth endorsement and appearance‐based cues, not just case evidence. Interventions should therefore target these underlying beliefs. In legal, forensic, and educational settings, training should address how rape myths and attractiveness stereotypes bias judgements of credibility and responsibility. Prevention efforts should challenge misconceptions about consent and victimhood while reducing reliance on extra‐evidentiary cues. The cross‐cultural findings further suggest that interventions should be tailored to local belief systems: whereas RMA explained differences between the Hungarian and US samples, it did not account for elevated victim blaming in the Turkish sample, indicating that factors such as honour norms and gender‐role beliefs may also be important. Finally, public communication and media reporting should avoid reinforcing appearance‐based stereotypes and instead promote evidence‐based evaluations of sexual assault allegations.

### 4.2 Limitations and Future Directions

This study was not preregistered. However, to enhance transparency and reproducibility, we have created a public Open Science Framework (OSF) repository containing all relevant study materials, data and analysis‐related information (https://doi.org/10.17605/OSF.IO/TDH6U).

Several limitations should be considered. First, the national samples differed in age and sex composition, which may complicate cross‐cultural comparisons. Although supplementary analyses indicated that age did not alter the mediation results, some national differences may still reflect demographic variation rather than cultural influences. Future research should employ age‐ and sex‐matched samples or more comprehensive demographic controls.

Second, ambiguity and cue weighting were inferred rather than directly measured. Although the vignettes were designed to approximate ambiguous judgement contexts, future studies should manipulate ambiguity and assess cue utilisation more directly (e.g., process‐tracing or attention‐based methods; Süssenbach et al. 2012).

Third, the design may have increased the salience of attractiveness. Because participants rated facial attractiveness immediately before completing the victim‐blame task, some effects may reflect demand characteristics. Future studies should include filler tasks, temporal delays, or suspicion probes.

Fourth, although the IRMA demonstrated excellent reliability, the Hungarian and Turkish versions were not independently validated, and traditional rape myth scales may be vulnerable to floor effects and social desirability bias. Future cross‐cultural studies should consider including measures such as the AMMSA, which may better capture subtle contemporary rape‐supportive beliefs (Gerger et al. 2007).

Fifth, the present study focused on mediation as a test of the schema account. However, moderation analyses examining whether RMA predicts victim blame more strongly under specific perpetrator‐victim attractiveness conditions may provide a more direct test of schema activation (Süssenbach et al. 2012).

Finally, the reversed attractiveness effect observed in Türkiye was not directly explained by measured variables. Future research should examine whether perceptions of sexual availability, modesty, attractiveness stereotypes, or victim respectability mediate these effects across cultures.

## 5 Conclusion

Physical attractiveness, rape myth acceptance (RMA) and cultural context jointly shaped victim blame judgements. Victim attractiveness influenced blame most strongly when paired with an attractive perpetrator, whereas RMA consistently predicted victim blaming and mediated the relationship between participant sex and blame. RMA also explained differences between the Hungarian and US samples but not the elevated victim blaming observed in the Turkish sample, suggesting that cultural contexts may rely on distinct ideological mechanisms when evaluating sexual assault allegations. Together, these findings underscore the importance of addressing both rape myth beliefs and appearance‐based stereotypes while recognising that victim blaming cannot be explained by a single cultural framework.

## Author Contributions

**Zoë Marleen Cheryl Brinkert:** visualization, formal analysis, investigation. **Ádám Putz:** conceptualization, methodology, data curation, supervision, resources, project administration, formal analysis, funding acquisition, writing – review and editing. **Amy E. Coren:** writing – original draft, supervision, conceptualization, investigation. **Ayşe Naz Hazal Sezen:** investigation, validation, writing – original draft.

## Funding

This study was supported by the University of Pécs (grant number: 009_2025_PTE_RK/8).

## Ethics Statement

All procedures performed in this study involving human participants were in accordance with the ethical standards of the Hungarian United Ethical Review Committee for Research in Psychology (reference number: 2018‐106; Date of approval: 3. December 2018) and the 1964 Helsinki Declaration and its later amendments or comparable ethical standards.

## Consent

Informed consent was obtained from all participants.

## Conflicts of Interest

The authors declare no conflicts of interest.

## Supporting information


**Data S1:** Supporting Information 1.

**Data S2:** Supporting Information 2.

**Data S3:** Supporting Information 3.

**Data S4:** Supporting Information 4.

**Data S5:** Supporting Information 5.

**Data S6:** Supporting Information 6.

**Data S7:** Supporting Information 7.

**Data S8:** Supporting Information 8.

**Data S9:** Supporting Information 9.

**Data S10:** Supporting Information 10.

**Data S11:** Supporting Information 11.

**Data S12:** Supporting Information 12.

**Data S13:** Supporting Information 13.

**Data S14:** Supporting Information 14.

**Data S15:** Supporting Information 15.

**Data S16:** Supporting Information 16.

## Data Availability

The datasets generated and/or analysed during the current study are available in the Open Science Framework (OSF) repository at https://doi.org/10.17605/OSF.IO/TDH6U.

## Endnotes

^1^
GII scores were obtained from the dataset of the United Nations Development Programme at the time of data collection (i.e., in 2018). *Note:* lower GII scores represent better performance regarding gender equality. Source: https://hdr.undp.org/data‐center/thematic‐composite‐indices/gender‐inequality‐index#/indicies/GII.

^2^
Less than ⅓ of the participants in these samples were non‐students. A Brown‐Forsythe analysis showed that there were no significant differences between students/non‐students and thus they were grouped together in the overall analysis.

^3^
To enhance transparency and reproducibility, all facial stimuli, pilot study ratings, and the statistical analyses used for stimulus selection are publicly available through the project's Open Science Framework (OSF) repository: https://doi.org/10.17605/OSF.IO/TDH6U.

^4^
As there were no standardized Hungarian or Turkish language versions of the IRMA available at the time, the original English language questionnaire was translated and then reverse‐translated into English to ensure the accuracy of the translation.
