# Supplementary material for: When Appearances Deceive: Rape Myth Schemas Influence Attractiveness Effects Across Cultures
Source: Int J Psychol. 2026 Aug 2;61(5):e70256. doi: 10.1002/ijop.70256 (PMC13429343; doi:10.1002/ijop.70256)
Supplement: Supplementary file 1 — Data S1: Supporting Information 1. [file IJOP-61-e70256-s008.pdf]

GLM Mediation Model (HUN sample)

Models Info

Mediators Models		
Full Model	m1	SUM_IRMAS ~ Sex
Indirect Effects	m2	AVG_AA_blame ~ SUM_IRMAS + Sex
	IE 1	Sex ⇒ SUM_IRMAS ⇒ AVG_AA_blame
Sample size	N	282

Path Model

Statistical Diagram

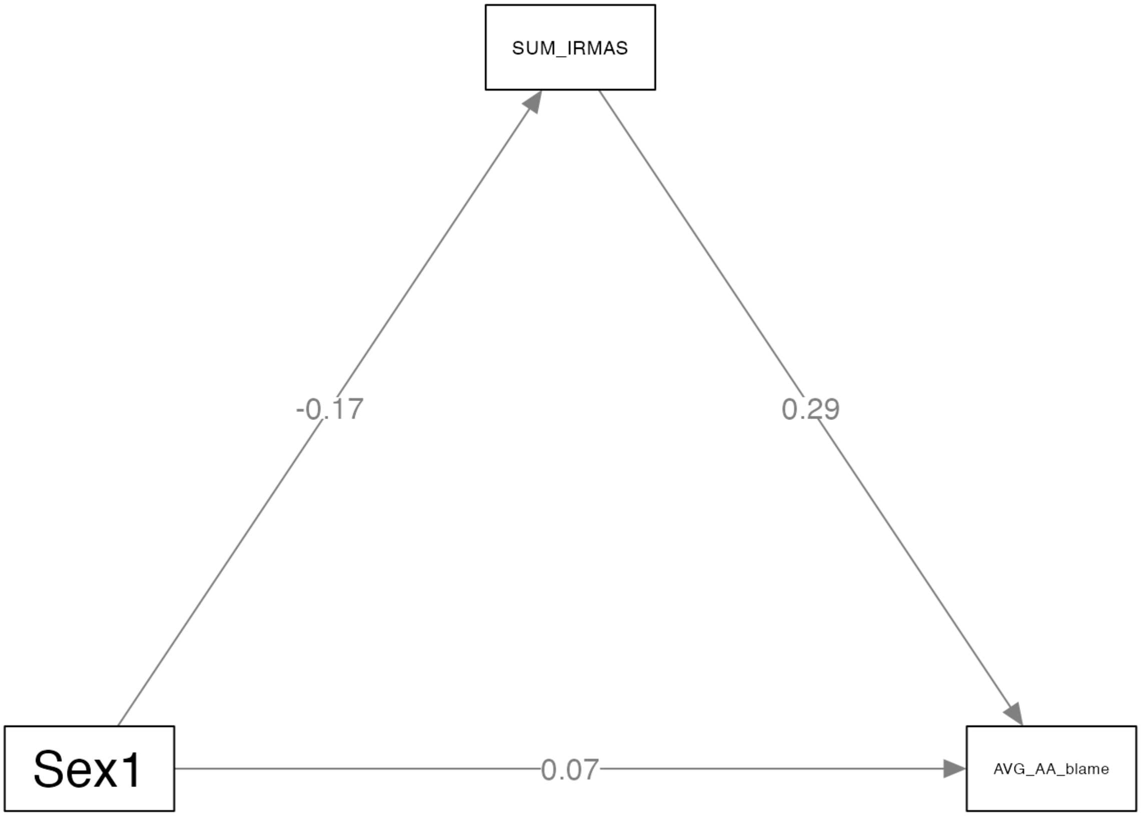

Diagram notes
Categorical independent variables (factors) are represented by contrast indicators
For variable **Sex** the contrasts are: Sex1 = Female - Male

Mediation

Indirect and Total Effects

Type	Effect	Estimate	SE	95% C.I. (a)		β	z	p
				Lower	Upper			
Indirect	Sex1 ⇒ SUM_IRMAS ⇒ AVG_AA_blame	-0.1498	0.05880	-0.26509	-0.0346	-0.0505	-2.548	.011
Component	Sex1 ⇒ SUM_IRMAS	-14.6317	4.95304	-24.33952	-4.9240	-0.1733	-2.954	.003
	SUM_IRMAS ⇒ AVG_AA_blame	0.0102	0.00203	0.00626	0.0142	0.2917	5.038	<.001
Direct	Sex1 ⇒ AVG_AA_blame	0.2071	0.17167	-0.12939	0.5436	0.0698	1.206	.228
Total	Sex1 ⇒ AVG_AA_blame	0.0572	0.17684	-0.28935	0.4038	0.0193	0.324	.746

Note. Confidence intervals computed with method: Standard (Delta method)

Note. Betas are completely standardized effect sizes
