# Supplementary material for: When Appearances Deceive: Rape Myth Schemas Influence Attractiveness Effects Across Cultures
Source: Int J Psychol. 2026 Aug 2;61(5):e70256. doi: 10.1002/ijop.70256 (PMC13429343; doi:10.1002/ijop.70256)
Supplement: Supplementary file 2 — Data S2: Supporting Information 2. [file IJOP-61-e70256-s005.pdf]

GLM Mediation Analysis (HUN sample)

Models Info

Mediators Models		
Full Model	m1	SUM_IRMAS ~ Sex
Indirect Effects	m2	AVG_AUA_B ~ SUM_IRMAS + Sex
	IE 1	Sex ⇒ SUM_IRMAS ⇒ AVG_AUA_B
Sample size	N	282

Path Model

Statistical Diagram

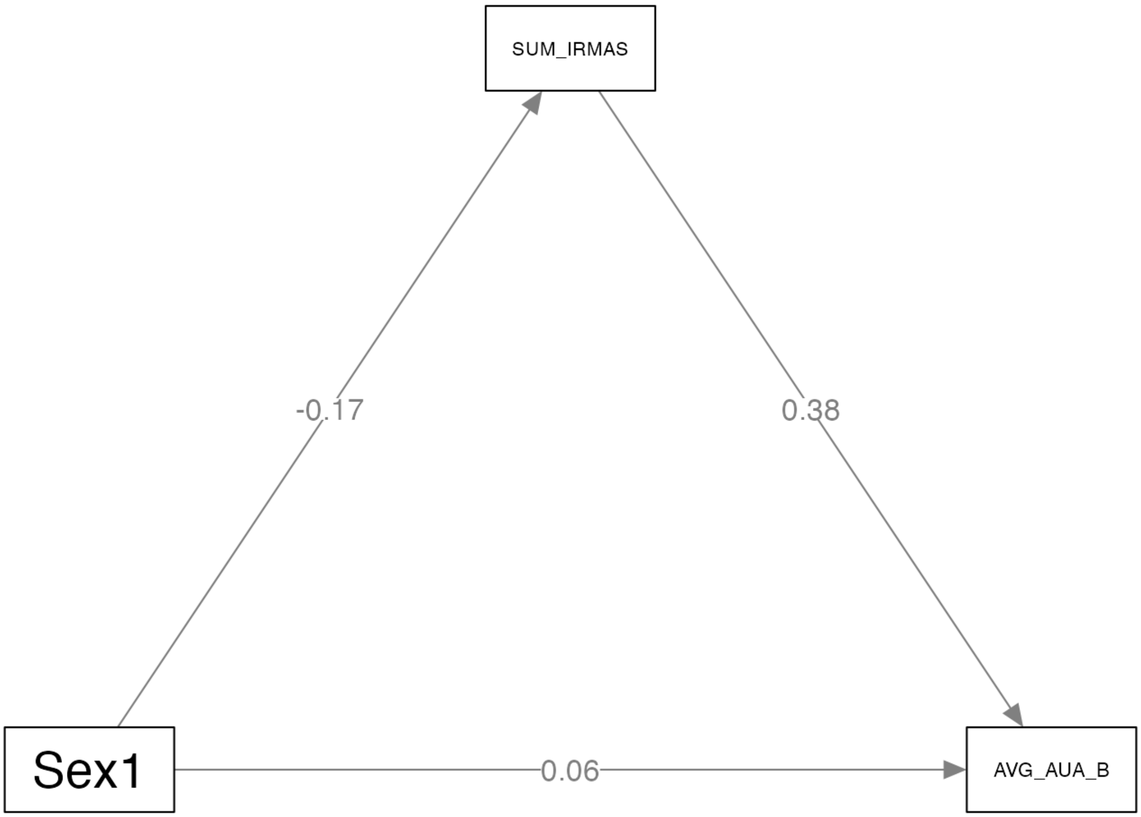

Diagram notes
Categorical independent variables (factors) are represented by contrast indicators
For variable **Sex** the contrasts are: Sex1 = Female - Male

Mediation

Indirect and Total Effects

Type	Effect	Estimate	SE	95% C.I. (a)		β	z	p
				Lower	Upper			
Indirect	Sex1 ⇒ SUM_IRMAS ⇒ AVG_AUA_B	-0.2225	0.08221	-0.3837	-0.0614	-0.06572	-2.707	.007
Component	Sex1 ⇒ SUM_IRMAS	-14.6317	4.95304	-24.3395	-4.9240	-0.17325	-2.954	.003
	SUM_IRMAS ⇒ AVG_AUA_B	0.0152	0.00225	0.0108	0.0196	0.37931	6.763	<.001
Direct	Sex1 ⇒ AVG_AUA_B	0.1912	0.18992	-0.1811	0.5634	0.05646	1.007	.314
Total	Sex1 ⇒ AVG_AUA_B	-0.0314	0.20201	-0.4273	0.3646	-0.00926	-0.155	.877

Note. Confidence intervals computed with method: Standard (Delta method)

Note. Betas are completely standardized effect sizes
