# Supplementary material for: When Appearances Deceive: Rape Myth Schemas Influence Attractiveness Effects Across Cultures
Source: Int J Psychol. 2026 Aug 2;61(5):e70256. doi: 10.1002/ijop.70256 (PMC13429343; doi:10.1002/ijop.70256)
Supplement: Supplementary file 3 — Data S3: Supporting Information 3. [file IJOP-61-e70256-s016.pdf]

GLM Mediation Analysis (HUN sample)

Models Info

Mediators Models		
Full Model	m1	SUM_IRMAS ~ Sex
Indirect Effects	m2	AVG_UAA_B ~ SUM_IRMAS + Sex
	IE 1	Sex ⇒ SUM_IRMAS ⇒ AVG_UAA_B
Sample size	N	282

Path Model

Statistical Diagram

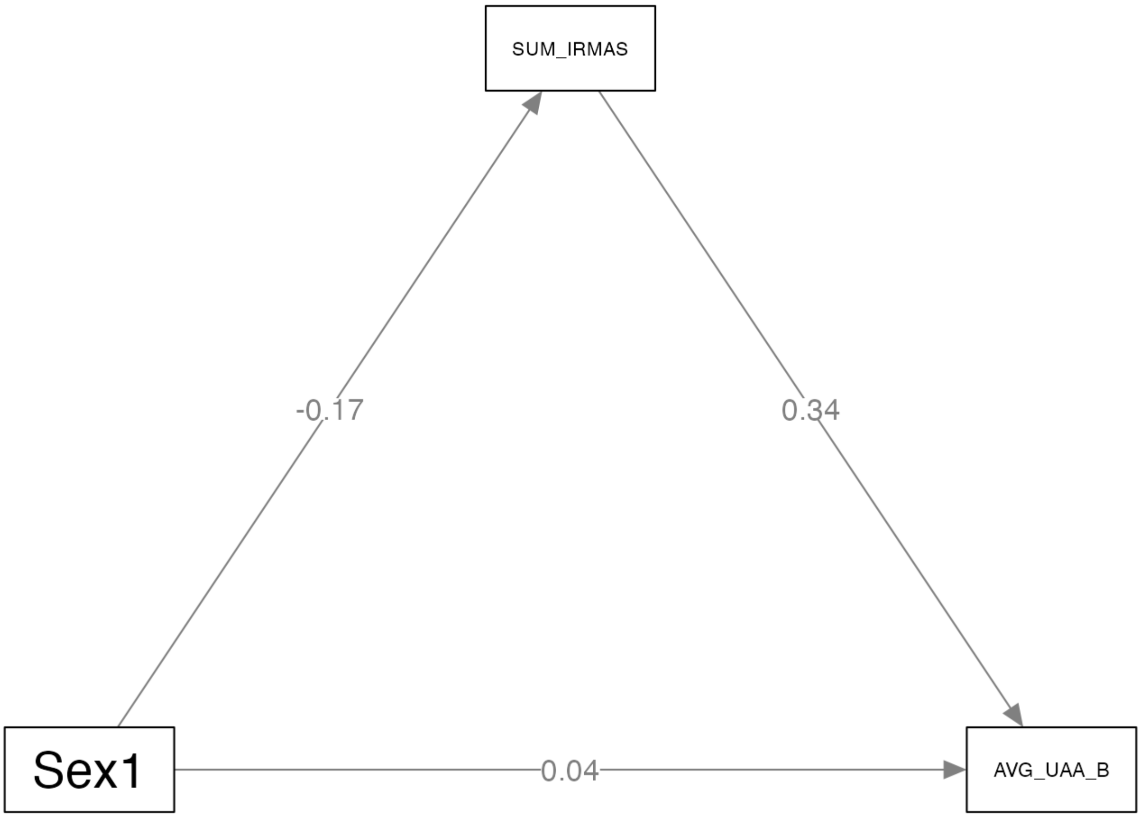

Diagram notes
Categorical independent variables (factors) are represented by contrast indicators
For variable **Sex** the contrasts are: Sex1 = Female - Male

Mediation

Indirect and Total Effects

Type	Effect	Estimate	SE	95% C.I. (a)		β	z	p
				Lower	Upper			
Indirect	Sex1 ⇒ SUM_IRMAS ⇒ AVG_UAA_B	-0.1694	0.06417	-0.29521	-0.0437	-0.0582	-2.640	.008
Component	Sex1 ⇒ SUM_IRMAS	-14.6317	4.95304	-24.33952	-4.9240	-0.1733	-2.954	.003
	SUM_IRMAS ⇒ AVG_UAA_B	0.0116	0.00197	0.00772	0.0154	0.3358	5.885	<.001
Direct	Sex1 ⇒ AVG_UAA_B	0.1261	0.16616	-0.19959	0.4518	0.0433	0.759	.448
Total	Sex1 ⇒ AVG_UAA_B	-0.0434	0.17372	-0.38383	0.2971	-0.0149	-0.250	.803

Note. Confidence intervals computed with method: Standard (Delta method)

Note. Betas are completely standardized effect sizes
