# Supplementary material for: When Appearances Deceive: Rape Myth Schemas Influence Attractiveness Effects Across Cultures
Source: Int J Psychol. 2026 Aug 2;61(5):e70256. doi: 10.1002/ijop.70256 (PMC13429343; doi:10.1002/ijop.70256)
Supplement: Supplementary file 4 — Data S4: Supporting Information 4. [file IJOP-61-e70256-s003.pdf]

GLM Mediation Analysis (HUN sample)

Models Info

Mediators Models		
Full Model	m1	SUM_IRMAS ~ Sex
Indirect Effects	m2	AVG_UAUA_B ~ SUM_IRMAS + Sex
	IE 1	Sex ⇒ SUM_IRMAS ⇒ AVG_UAUA_B
Sample size	N	282

Path Model

Statistical Diagram

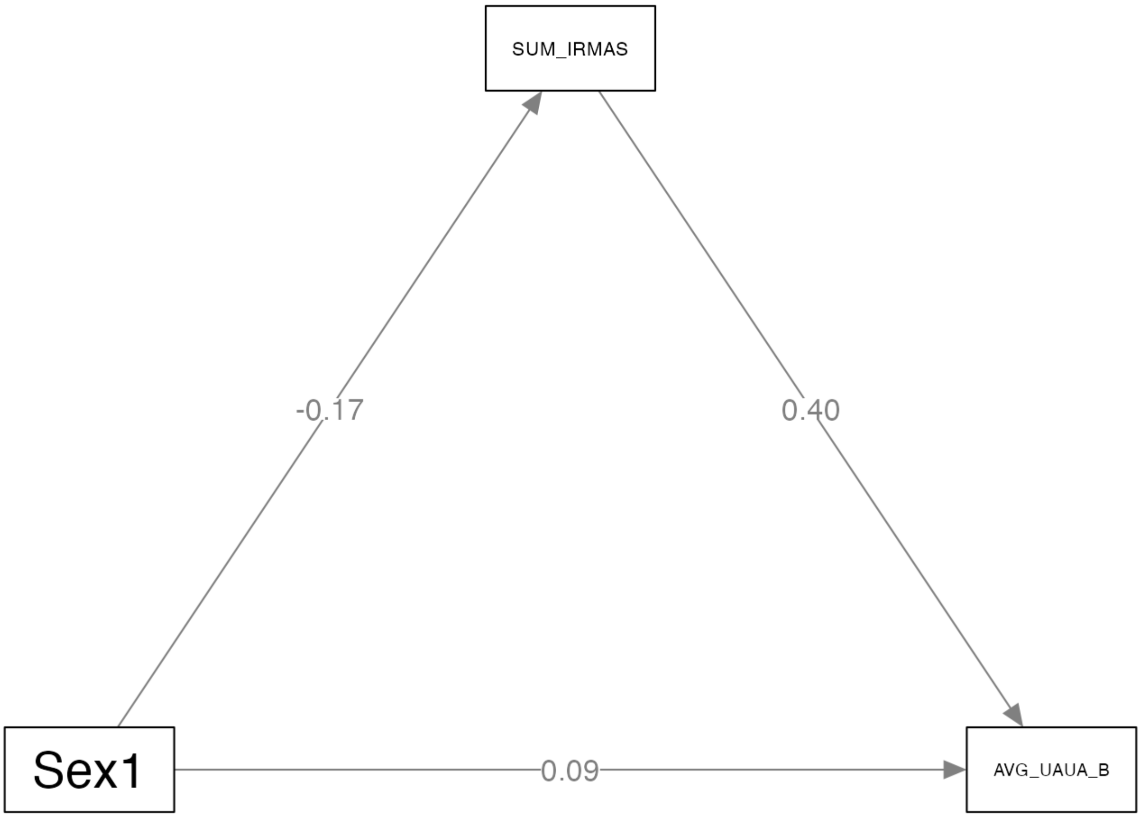

Diagram notes
Categorical independent variables (factors) are represented by contrast indicators
For variable **Sex** the contrasts are: Sex1 = Female - Male

Mediation

Indirect and Total Effects

Type	Effect	Estimate	SE	95% C.I. (a)		β	z	p
				Lower	Upper			
Indirect	Sex1 ⇒ SUM_IRMAS ⇒ AVG_UAUA_B	-0.2062	0.07538	-0.3540	-0.0585	-0.0697	-2.736	.006
Component	Sex1 ⇒ SUM_IRMAS	-14.6317	4.95304	-24.3395	-4.9240	-0.1733	-2.954	.003
	SUM_IRMAS ⇒ AVG_UAUA_B	0.0141	0.00194	0.0103	0.0179	0.4025	7.253	<.001
Direct	Sex1 ⇒ AVG_UAUA_B	0.2679	0.16412	-0.0538	0.5895	0.0906	1.632	.103
Total	Sex1 ⇒ AVG_UAUA_B	0.0617	0.17638	-0.2840	0.4074	0.0208	0.350	.727

Note. Confidence intervals computed with method: Standard (Delta method)

Note. Betas are completely standardized effect sizes
