# Supplementary material for: When Appearances Deceive: Rape Myth Schemas Influence Attractiveness Effects Across Cultures
Source: Int J Psychol. 2026 Aug 2;61(5):e70256. doi: 10.1002/ijop.70256 (PMC13429343; doi:10.1002/ijop.70256)
Supplement: Supplementary file 5 — Data S5: Supporting Information 5. [file IJOP-61-e70256-s002.pdf]

GLM Mediation Model (TUR sample)

Models Info

Mediators Models		
Full Model	m1	SUM_IRMAS ~ Sex
Indirect Effects	m2	AVG_AA_blame ~ SUM_IRMAS + Sex
	IE 1	Sex ⇒ SUM_IRMAS ⇒ AVG_AA_blame
Sample size	N	399

Path Model

Statistical Diagram

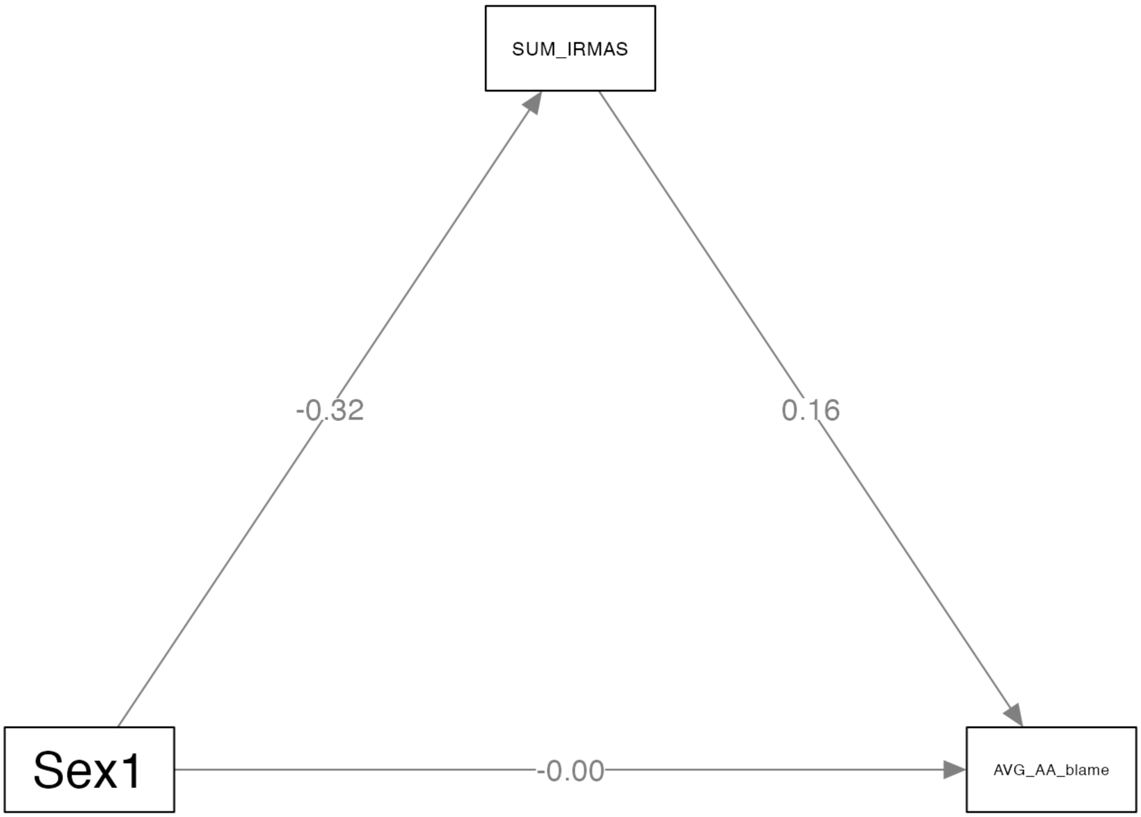

Diagram notes
Categorical independent variables (factors) are represented by contrast indicators
For variable **Sex** the contrasts are: Sex1 = Female - Male

Mediation

Indirect and Total Effects

Type	Effect	Estimate	SE	95% C.I. (a)		β	z	p
				Lower	Upper			
Indirect	Sex1 ⇒ SUM_IRMAS ⇒ AVG_AA_blame	-0.2526	0.09283	-0.43452	-0.0706	-0.04951	-2.7209	.007
Component	Sex1 ⇒ SUM_IRMAS	-17.8380	2.65417	-23.04008	-12.6359	-0.31889	-6.7207	<.001
	SUM_IRMAS ⇒ AVG_AA_blame	0.0142	0.00476	0.00483	0.0235	0.15525	2.9757	.003
Direct	Sex1 ⇒ AVG_AA_blame	-0.0157	0.26618	-0.53736	0.5060	-0.00307	-0.0589	.953
Total	Sex1 ⇒ AVG_AA_blame	-0.2683	0.25538	-0.76879	0.2323	-0.05258	-1.0504	.294

Note. Confidence intervals computed with method: Standard (Delta method)

Note. Betas are completely standardized effect sizes
