# Supplementary material for: When Appearances Deceive: Rape Myth Schemas Influence Attractiveness Effects Across Cultures
Source: Int J Psychol. 2026 Aug 2;61(5):e70256. doi: 10.1002/ijop.70256 (PMC13429343; doi:10.1002/ijop.70256)
Supplement: Supplementary file 6 — Data S6: Supporting Information 6. [file IJOP-61-e70256-s010.pdf]

GLM Mediation Analysis (TUR sample)

Models Info

Mediators Models		
Full Model	m1	SUM_IRMAS ~ Sex
Indirect Effects	m2	AVG_AUA_B ~ SUM_IRMAS + Sex
	IE 1	Sex ⇒ SUM_IRMAS ⇒ AVG_AUA_B
Sample size	N	399

Path Model

Statistical Diagram

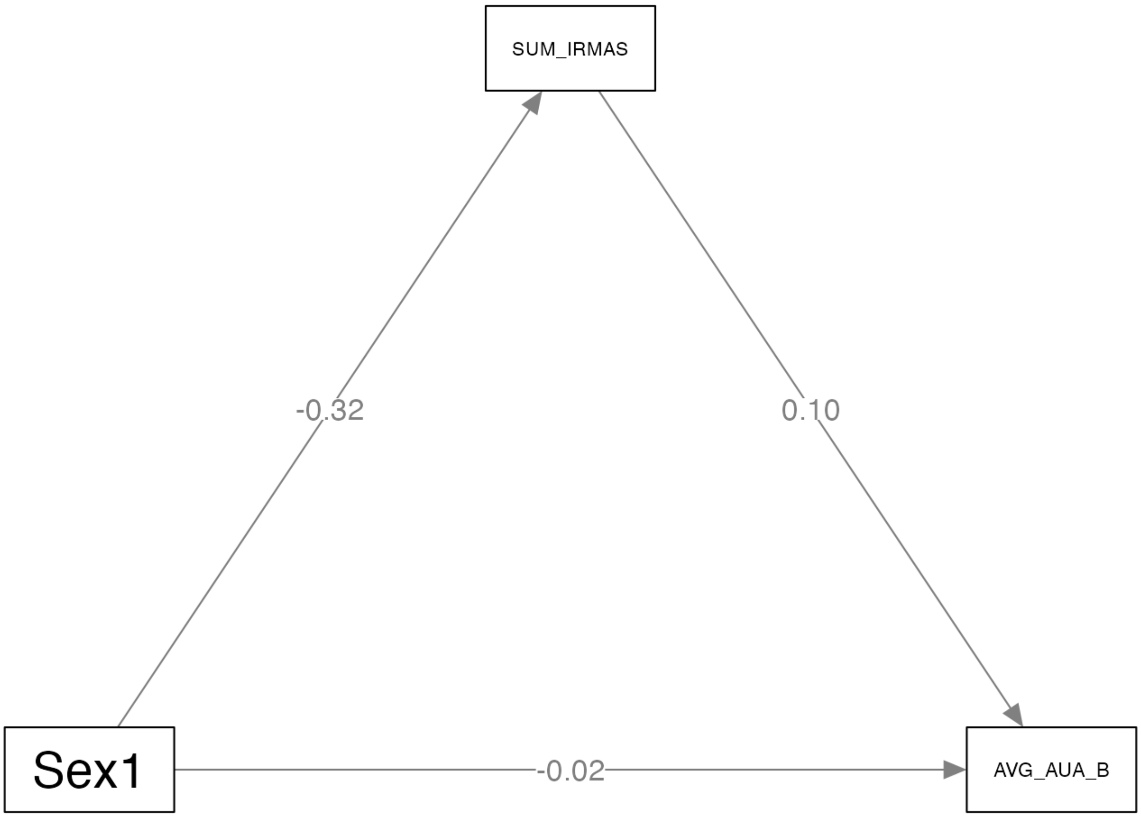

Diagram notes
Categorical independent variables (factors) are represented by contrast indicators
For variable **Sex** the contrasts are: Sex1 = Female - Male

Mediation

Indirect and Total Effects

Type	Effect	Estimate	SE	95% C.I. (a) Lower	Upper	β	z	p
Indirect	Sex1 ⇒ SUM_IRMAS ⇒ AVG_AUA_B	-0.16874	0.08885	-0.343	0.00540	-0.0331	-1.899	.058
Component	Sex1 ⇒ SUM_IRMAS	-17.83800	2.65417	-23.040	-12.63592	-0.3189	-6.721	<.001
	SUM_IRMAS ⇒ AVG_AUA_B	0.00946	0.00478	9.51e-5	0.01882	0.1039	1.980	.048
Direct	Sex1 ⇒ AVG_AUA_B	-0.08099	0.26726	-0.605	0.44282	-0.0159	-0.303	.762
Total	Sex1 ⇒ AVG_AUA_B	-0.24973	0.25486	-0.749	0.24979	-0.0491	-0.980	.327

Note. Confidence intervals computed with method: Standard (Delta method)

Note. Betas are completely standardized effect sizes
