# Supplementary material for: When Appearances Deceive: Rape Myth Schemas Influence Attractiveness Effects Across Cultures
Source: Int J Psychol. 2026 Aug 2;61(5):e70256. doi: 10.1002/ijop.70256 (PMC13429343; doi:10.1002/ijop.70256)
Supplement: Supplementary file 7 — Data S7: Supporting Information 7. [file IJOP-61-e70256-s015.pdf]

GLM Mediation Analysis (TUR sample)

Models Info

Mediators Models		
Full Model	m1	SUM_IRMAS ~ Sex
Indirect Effects	m2	AVG_UAA_B ~ SUM_IRMAS + Sex
	IE 1	Sex ⇒ SUM_IRMAS ⇒ AVG_UAA_B
Sample size	N	399

Path Model

Statistical Diagram

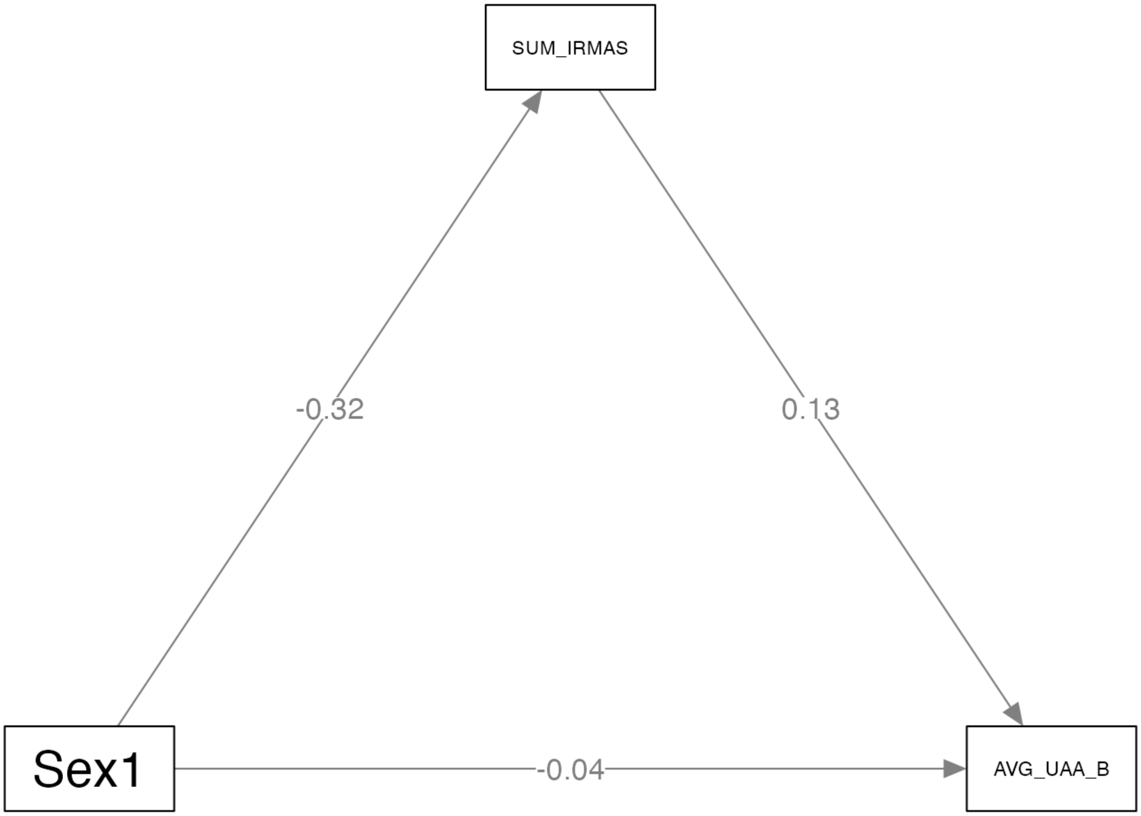

Diagram notes
Categorical independent variables (factors) are represented by contrast indicators
For variable **Sex** the contrasts are: Sex1 = Female - Male

Mediation

Indirect and Total Effects

Type	Effect	Estimate	SE	95% C.I. (a) Lower	Upper	β	z	p
Indirect	Sex1 ⇒ SUM_IRMAS ⇒ AVG_UAA_B	-0.2125	0.08851	-0.38594	-0.0390	-0.0428	-2.400	.016
Component	Sex1 ⇒ SUM_IRMAS	-17.8380	2.65417	-23.04008	-12.6359	-0.3189	-6.721	<.001
	SUM_IRMAS ⇒ AVG_UAA_B	0.0119	0.00463	0.00283	0.0210	0.1342	2.570	.010
Direct	Sex1 ⇒ AVG_UAA_B	-0.1904	0.25924	-0.69853	0.3177	-0.0384	-0.735	.463
Total	Sex1 ⇒ AVG_UAA_B	-0.4029	0.24804	-0.88905	0.0833	-0.0811	-1.624	.104

Note. Confidence intervals computed with method: Standard (Delta method)

Note. Betas are completely standardized effect sizes
