# Supplementary material for: When Appearances Deceive: Rape Myth Schemas Influence Attractiveness Effects Across Cultures
Source: Int J Psychol. 2026 Aug 2;61(5):e70256. doi: 10.1002/ijop.70256 (PMC13429343; doi:10.1002/ijop.70256)
Supplement: Supplementary file 8 — Data S8: Supporting Information 8. [file IJOP-61-e70256-s012.pdf]

GLM Mediation Analysis (TUR sample)

Models Info

Mediators Models		
Full Model	m1	SUM_IRMAS ~ Sex
Indirect Effects	m2	AVG_UAUA_B ~ SUM_IRMAS + Sex
	IE 1	Sex ⇒ SUM_IRMAS ⇒ AVG_UAUA_B
Sample size	N	399

Path Model

Statistical Diagram

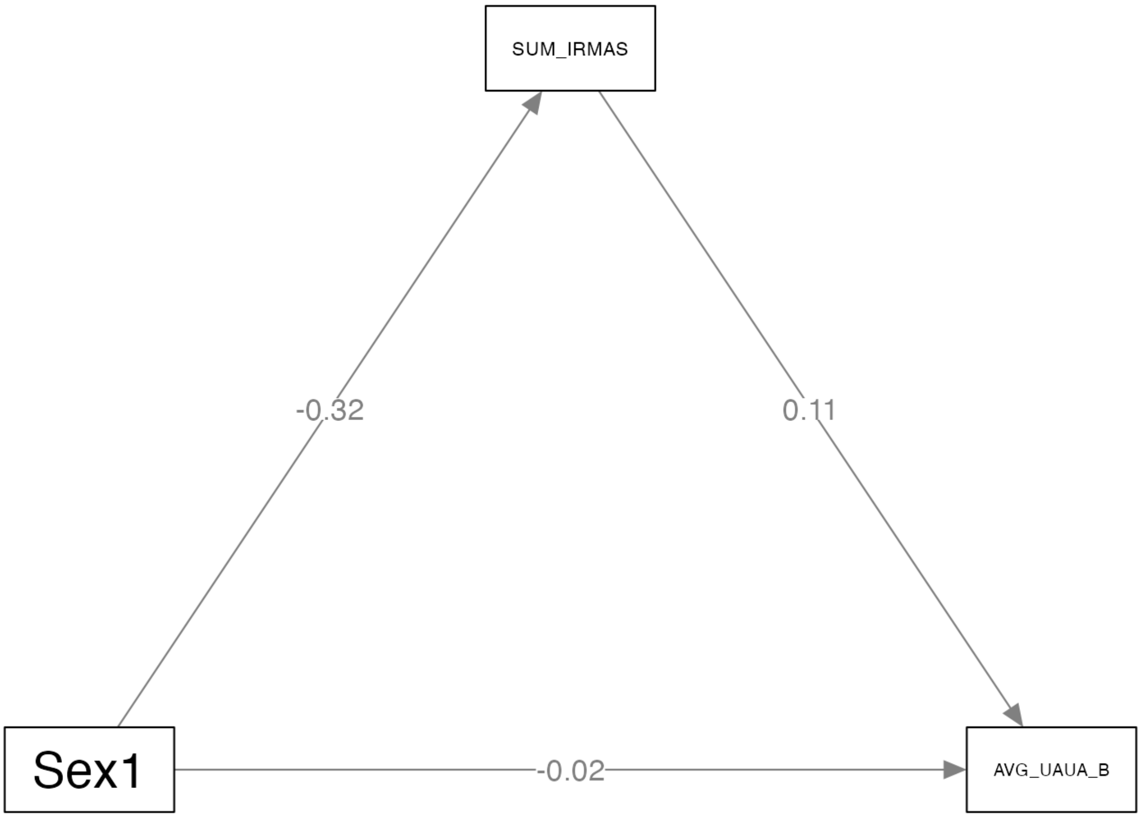

Diagram notes
Categorical independent variables (factors) are represented by contrast indicators
For variable **Sex** the contrasts are: Sex1 = Female - Male

Mediation

Indirect and Total Effects

Type	Effect	Estimate	SE	95% C.I. (a)		β	z	p
				Lower	Upper			
Indirect	Sex1 ⇒ SUM_IRMAS ⇒ AVG_UAUA_B	-0.1859	0.08971	-0.36176	-0.0101	-0.0364	-2.072	.038
Component	Sex1 ⇒ SUM_IRMAS	-17.8380	2.65417	-23.04008	-12.6359	-0.3189	-6.721	<.001
	SUM_IRMAS ⇒ AVG_UAUA_B	0.0104	0.00478	0.00105	0.0198	0.1142	2.179	.029
Direct	Sex1 ⇒ AVG_UAUA_B	-0.0790	0.26762	-0.60355	0.4455	-0.0155	-0.295	.768
Total	Sex1 ⇒ AVG_UAUA_B	-0.2649	0.25548	-0.76567	0.2358	-0.0519	-1.037	.300

Note. Confidence intervals computed with method: Standard (Delta method)

Note. Betas are completely standardized effect sizes
