# Supplementary material for: When Appearances Deceive: Rape Myth Schemas Influence Attractiveness Effects Across Cultures
Source: Int J Psychol. 2026 Aug 2;61(5):e70256. doi: 10.1002/ijop.70256 (PMC13429343; doi:10.1002/ijop.70256)
Supplement: Supplementary file 9 — Data S9: Supporting Information 9. [file IJOP-61-e70256-s007.pdf]

GLM Mediation Model (US sample)

Models Info

Mediators Models		
Full Model	m1	SUM_IRMAS ~ Sex
Indirect Effects	m2	AVG_AA_blame ~ SUM_IRMAS + Sex
	IE 1	Sex ⇒ SUM_IRMAS ⇒ AVG_AA_blame
Sample size	N	298

Path Model

Statistical Diagram

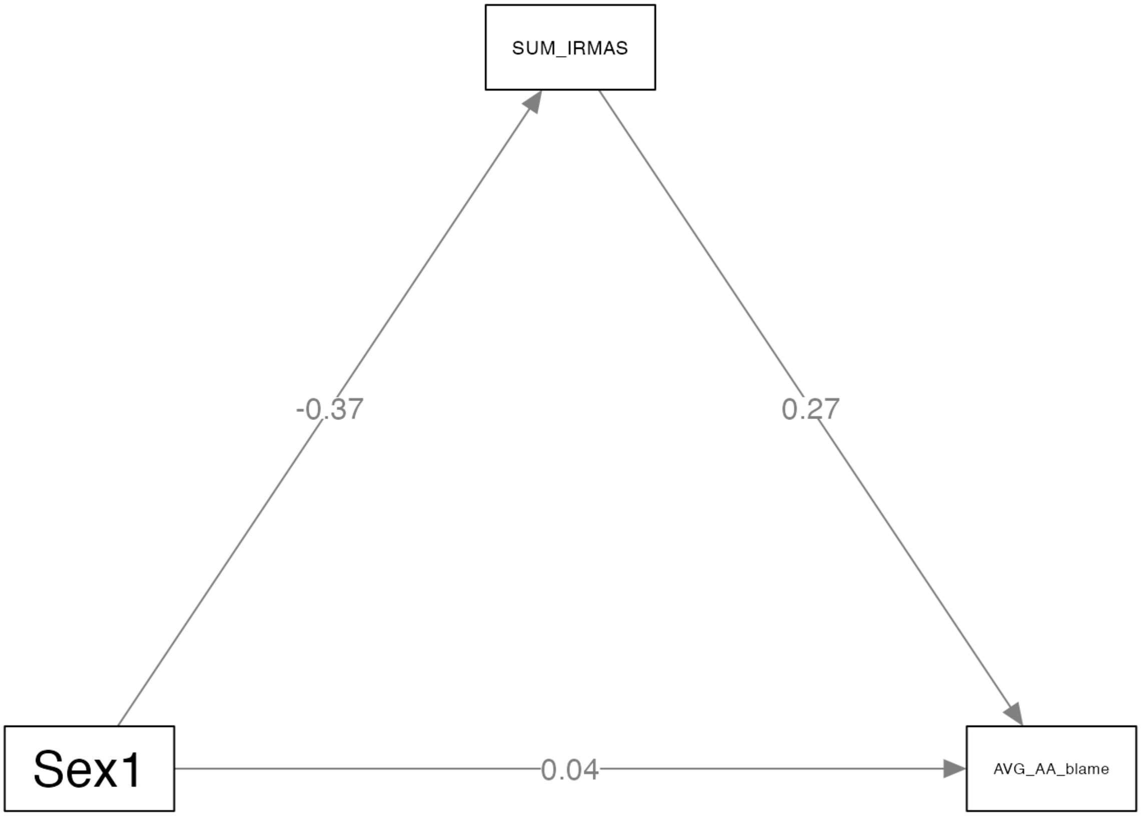

Diagram notes
Categorical independent variables (factors) are represented by contrast indicators
For variable **Sex** the contrasts are: Sex1 = Female - Male

Mediation

Indirect and Total Effects

Type	Effect	Estimate	SE	95% C.I. (a)		β	z	p
				Lower	Upper			
Indirect	Sex1 ⇒ SUM_IRMAS ⇒ AVG_AA_blame	-0.2937	0.07799	-0.44658	-0.1409	-0.0996	-3.766	<.001
Component	Sex1 ⇒ SUM_IRMAS	-28.6340	4.21192	-36.88919	-20.3788	-0.3664	-6.798	<.001
	SUM_IRMAS ⇒ AVG_AA_blame	0.0103	0.00227	0.00581	0.0147	0.2719	4.524	<.001
Direct	Sex1 ⇒ AVG_AA_blame	0.1128	0.17719	-0.23450	0.4601	0.0383	0.637	.524
Total	Sex1 ⇒ AVG_AA_blame	-0.1809	0.17072	-0.51555	0.1537	-0.0614	-1.060	.289

Note. Confidence intervals computed with method: Standard (Delta method)

Note. Betas are completely standardized effect sizes
