# Supplementary material for: When Appearances Deceive: Rape Myth Schemas Influence Attractiveness Effects Across Cultures
Source: Int J Psychol. 2026 Aug 2;61(5):e70256. doi: 10.1002/ijop.70256 (PMC13429343; doi:10.1002/ijop.70256)
Supplement: Supplementary file 10 — Data S10: Supporting Information 10. [file IJOP-61-e70256-s014.pdf]

GLM Mediation Analysis (US sample)

Models Info

Mediators Models		
Full Model	m1	SUM_IRMAS ~ Sex
Indirect Effects	m2	AVG_AUA_B ~ SUM_IRMAS + Sex
	IE 1	Sex ⇒ SUM_IRMAS ⇒ AVG_AUA_B
Sample size	N	298

Path Model

Statistical Diagram

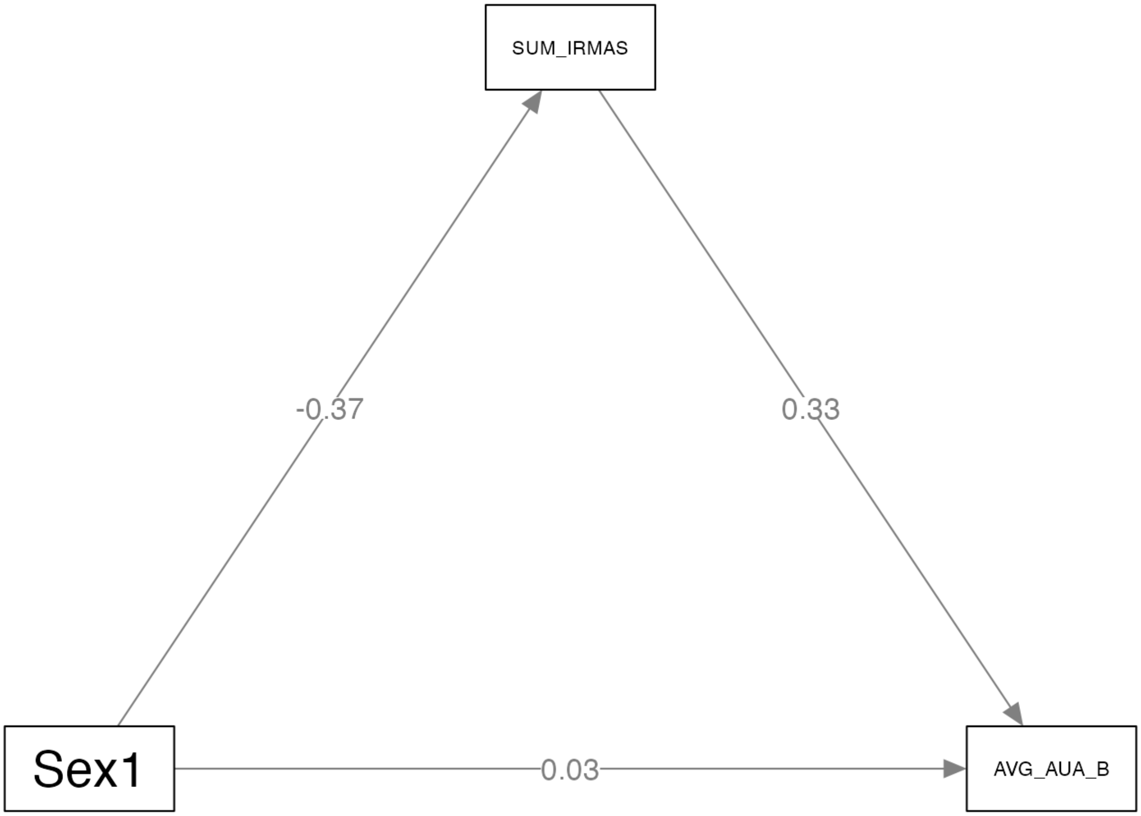

Diagram notes
Categorical independent variables (factors) are represented by contrast indicators
For variable **Sex** the contrasts are: Sex1 = Female - Male

Mediation

Indirect and Total Effects

Type	Effect	Estimate	SE	95% C.I. (a) Lower	Upper	β	z	p
Indirect	Sex1 ⇒ SUM_IRMAS ⇒ AVG_AUA_B	-0.3808	0.08863	-0.55455	-0.2071	-0.1200	-4.297	<.001
Component	Sex1 ⇒ SUM_IRMAS	-28.6340	4.21192	-36.88919	-20.3788	-0.3664	-6.798	<.001
	SUM_IRMAS ⇒ AVG_AUA_B	0.0133	0.00240	0.00860	0.0180	0.3274	5.545	<.001
Direct	Sex1 ⇒ AVG_AUA_B	0.0977	0.18743	-0.26968	0.4650	0.0308	0.521	.602
Total	Sex1 ⇒ AVG_AUA_B	-0.2832	0.18348	-0.64278	0.0765	-0.0892	-1.543	.123

Note. Confidence intervals computed with method: Standard (Delta method)

Note. Betas are completely standardized effect sizes
