# Supplementary material for: When Appearances Deceive: Rape Myth Schemas Influence Attractiveness Effects Across Cultures
Source: Int J Psychol. 2026 Aug 2;61(5):e70256. doi: 10.1002/ijop.70256 (PMC13429343; doi:10.1002/ijop.70256)
Supplement: Supplementary file 11 — Data S11: Supporting Information 11. [file IJOP-61-e70256-s001.pdf]

GLM Mediation Analysis (US sample)

Models Info

Mediators Models		
Full Model	m1	SUM_IRMAS ~ Sex
Indirect Effects	m2	AVG_UAA_B ~ SUM_IRMAS + Sex
	IE 1	Sex ⇒ SUM_IRMAS ⇒ AVG_UAA_B
Sample size	N	298

Path Model

Statistical Diagram

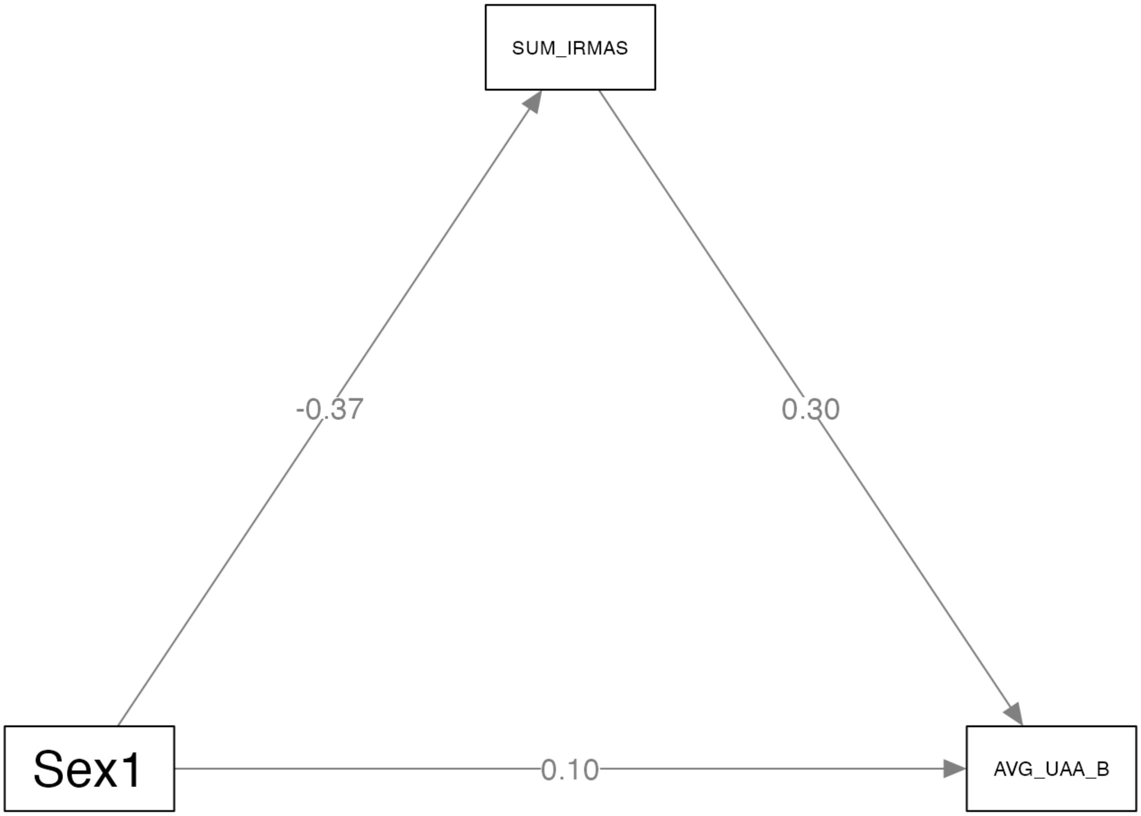

Diagram notes
Categorical independent variables (factors) are represented by contrast indicators
For variable **Sex** the contrasts are: Sex1 = Female - Male

Mediation

Indirect and Total Effects

Type	Effect	Estimate	SE	95% C.I. (a)		β	z	p
				Lower	Upper			
Indirect	Sex1 ⇒ SUM_IRMAS ⇒ AVG_UAA_B	-0.3289	0.08160	-0.48880	-0.1689	-0.10964	-4.030	<.001
Component	Sex1 ⇒ SUM_IRMAS	-28.6340	4.21192	-36.88919	-20.3788	-0.36643	-6.798	<.001
	SUM_IRMAS ⇒ AVG_UAA_B	0.0115	0.00230	0.00699	0.0160	0.29920	5.004	<.001
Direct	Sex1 ⇒ AVG_UAA_B	0.3089	0.17936	-0.04265	0.6604	0.10298	1.722	.085
Total	Sex1 ⇒ AVG_UAA_B	-0.0200	0.17405	-0.36109	0.3212	-0.00666	-0.115	.909

Note. Confidence intervals computed with method: Standard (Delta method)

Note. Betas are completely standardized effect sizes
