# Supplementary material for: When Appearances Deceive: Rape Myth Schemas Influence Attractiveness Effects Across Cultures
Source: Int J Psychol. 2026 Aug 2;61(5):e70256. doi: 10.1002/ijop.70256 (PMC13429343; doi:10.1002/ijop.70256)
Supplement: Supplementary file 12 — Data S12: Supporting Information 12. [file IJOP-61-e70256-s011.pdf]

GLM Mediation Analysis (US sample)

Models Info

Mediators Models		
Full Model	m1	SUM_IRMAS ~ Sex
Indirect Effects	m2	AVG_UAUA_B ~ SUM_IRMAS + Sex
	IE 1	Sex ⇒ SUM_IRMAS ⇒ AVG_UAUA_B
Sample size	N	298

Path Model

Statistical Diagram

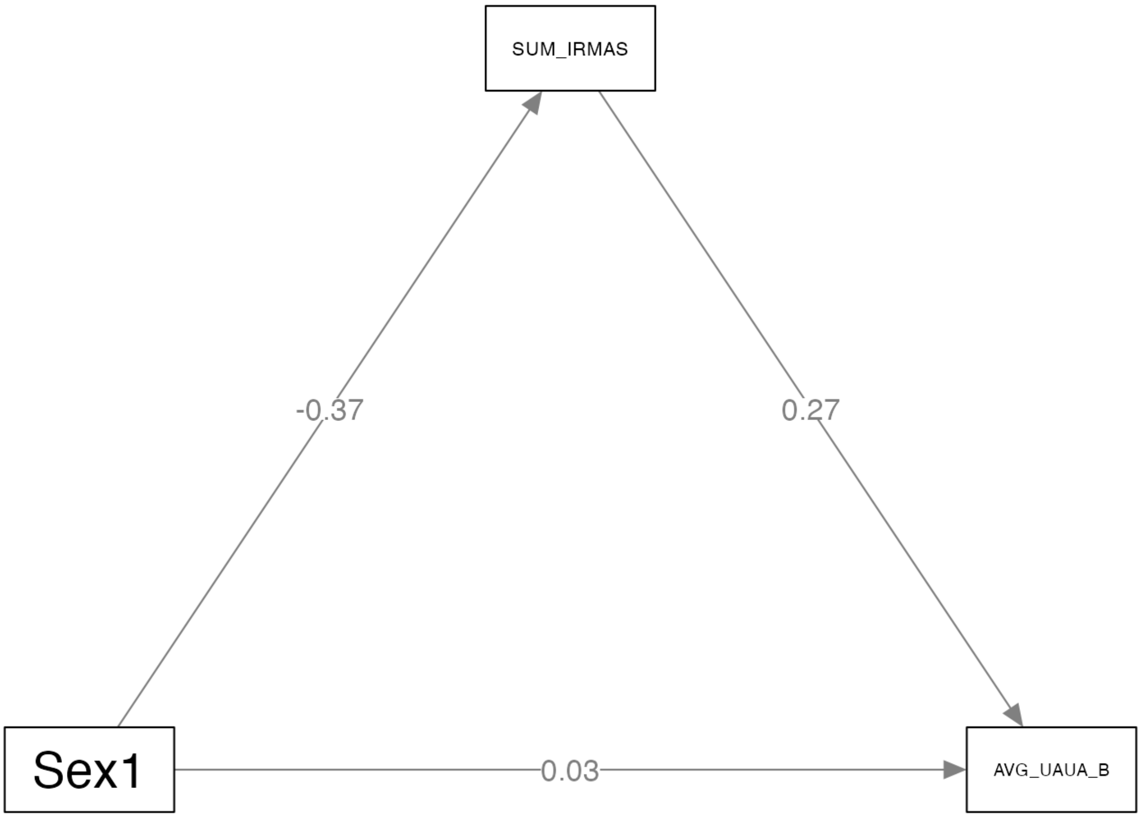

Diagram notes
Categorical independent variables (factors) are represented by contrast indicators
For variable **Sex** the contrasts are: Sex1 = Female - Male

Mediation

Indirect and Total Effects

Type	Effect	Estimate	SE	95% C.I. (a)		β	z	p
				Lower	Upper			
Indirect	Sex1 ⇒ SUM_IRMAS ⇒ AVG_UAUA_B	-0.2958	0.07928	-0.45120	-0.1404	-0.0984	-3.731	<.001
Component	Sex1 ⇒ SUM_IRMAS	-28.6340	4.21192	-36.88919	-20.3788	-0.3664	-6.798	<.001
	SUM_IRMAS ⇒ AVG_UAUA_B	0.0103	0.00231	0.00579	0.0149	0.2684	4.463	<.001
Direct	Sex1 ⇒ AVG_UAUA_B	0.0925	0.18087	-0.26196	0.4470	0.0308	0.512	.609
Total	Sex1 ⇒ AVG_UAUA_B	-0.2033	0.17412	-0.54454	0.1380	-0.0676	-1.167	.243

Note. Confidence intervals computed with method: Standard (Delta method)

Note. Betas are completely standardized effect sizes
