# Supplementary material for: When Appearances Deceive: Rape Myth Schemas Influence Attractiveness Effects Across Cultures
Source: Int J Psychol. 2026 Aug 2;61(5):e70256. doi: 10.1002/ijop.70256 (PMC13429343; doi:10.1002/ijop.70256)
Supplement: Supplementary file 13 — Data S13: Supporting Information 13. [file IJOP-61-e70256-s004.pdf]

GLM Mediation Analysis

Models Info

Mediators Models		
Full Model	m1	SUM_IRMAS ~ Nationality
Indirect Effects	m2	AVG_AA_blame ~ SUM_IRMAS + Nationality
	IE 1	Nationality ⇒ SUM_IRMAS ⇒ AVG_AA_blame
Sample size	N	979

Path Model

Statistical Diagram

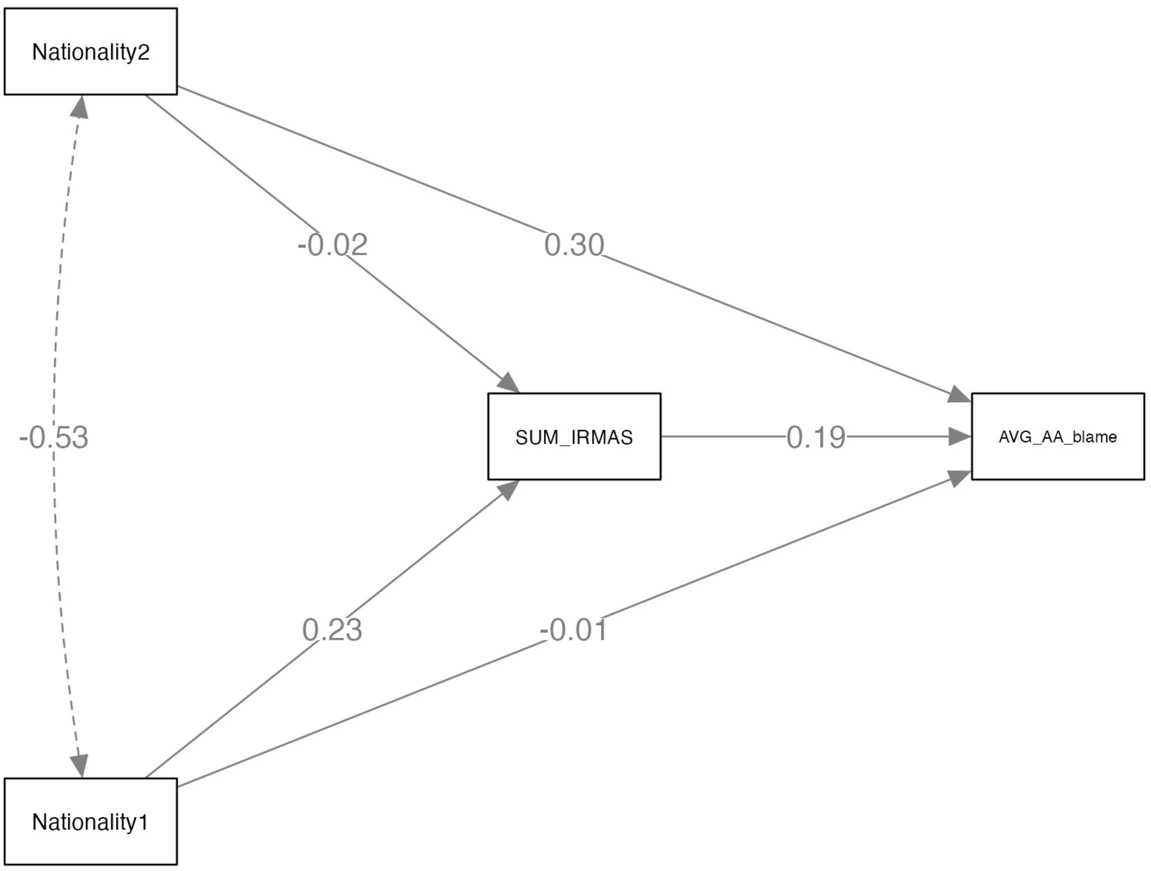

Diagram notes
Categorical independent variables (factors) are represented by contrast indicators
For variable **Nationality** the contrasts are: Nationality1 = HUN - US, Nationality2 = TUR - US

Mediation

Indirect and Total Effects

Type	Effect	Estimate	SE	95% C.I. (a)		β	z	p
				Lower	Upper			
Indirect	Nationality1 ⇒ SUM_IRMAS ⇒ AVG_AA_blame	0.1891	0.04324	0.10878	0.2863	0.04331	4.374	<.001
	Nationality2 ⇒ SUM_IRMAS ⇒ AVG_AA_blame	-0.0160	0.02780	-0.07712	0.0374	-0.00397	-0.574	.566
Component	Nationality1 ⇒ SUM_IRMAS	17.0655	2.70975	11.38530	22.9265	0.22991	6.298	<.001
	SUM_IRMAS ⇒ AVG_AA_blame	0.0111	0.00182	0.00733	0.0147	0.18839	6.080	<.001
	Nationality2 ⇒ SUM_IRMAS	-1.4400	2.49730	-6.45687	3.3680	-0.02105	-0.577	.564
Direct	Nationality1 ⇒ AVG_AA_blame	-0.0527	0.15763	-0.27662	0.1597	-0.01207	-0.334	.738
	Nationality2 ⇒ AVG_AA_blame	1.2269	0.14244	0.94487	1.5225	0.30489	8.613	<.001
Total	Nationality1 ⇒ AVG_AA_blame	0.1364	0.15751	-0.06859	0.3442	0.03125	0.866	.386
	Nationality2 ⇒ AVG_AA_blame	1.2110	0.14516	0.92447	1.5114	0.30093	8.342	<.001

Note. Confidence intervals computed with method: Bootstrap percentiles

Note. Betas are completely standardized effect sizes
