# Supplementary material for: When Appearances Deceive: Rape Myth Schemas Influence Attractiveness Effects Across Cultures
Source: Int J Psychol. 2026 Aug 2;61(5):e70256. doi: 10.1002/ijop.70256 (PMC13429343; doi:10.1002/ijop.70256)
Supplement: Supplementary file 14 — Data S14: Supporting Information 14. [file IJOP-61-e70256-s006.pdf]

GLM Mediation Analysis

Models Info

Mediators Models		
Full Model	m1	SUM_IRMAS ~ Nationality
Indirect Effects	m2	AVG_AUA_blame ~ SUM_IRMAS + Nationality
	IE 1	Nationality ⇒ SUM_IRMAS ⇒ AVG_AUA_blame
Sample size	N	979

Path Model

Statistical Diagram

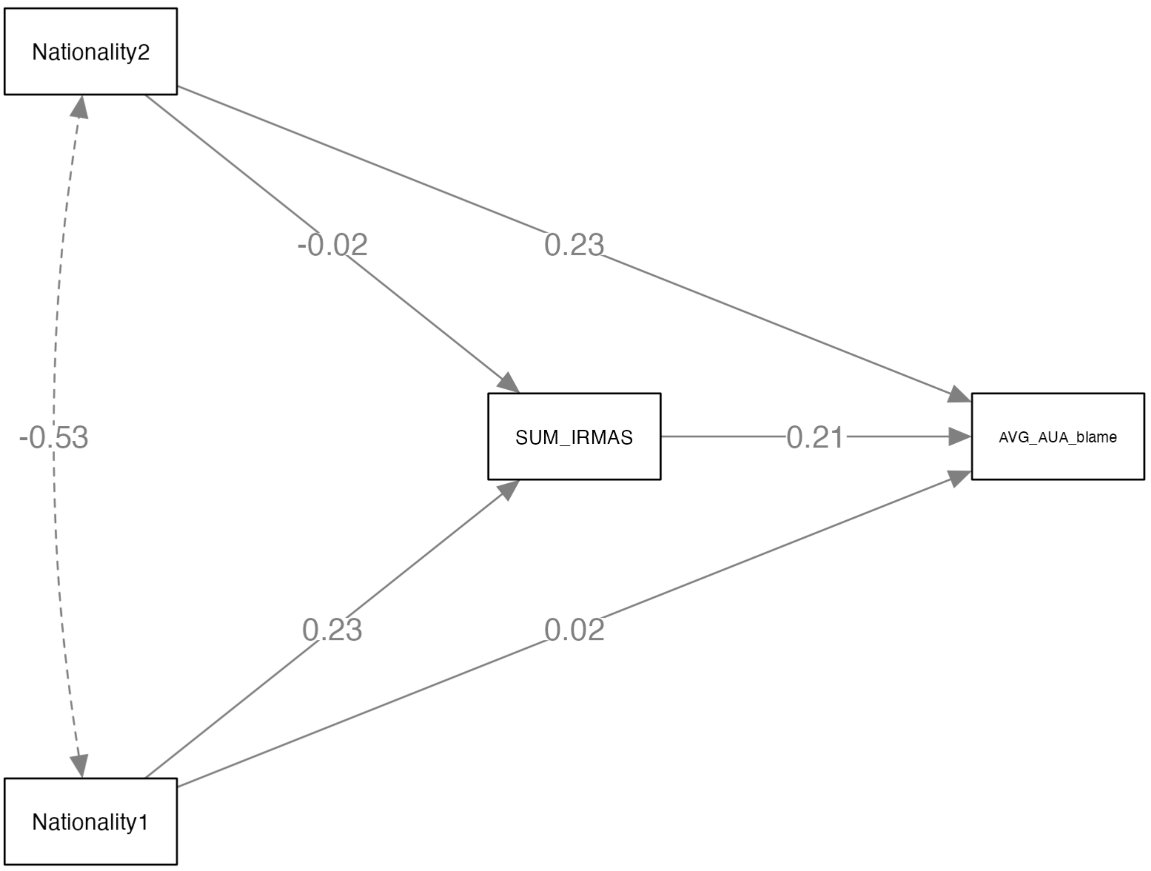

Diagram notes
Categorical independent variables (factors) are represented by contrast indicators
For variable **Nationality** the contrasts are: Nationality1 = HUN - US, Nationality2 = TUR - US

Mediation

Indirect and Total Effects

Type	Effect	Estimate	SE	95% C.I. (a)		β	z	p
				Lower	Upper			
Indirect	Nationality1 ⇒ SUM_IRMAS ⇒ AVG_AUA_blame	0.2162	0.04685	0.12985	0.3195	0.04922	4.615	<.001
	Nationality2 ⇒ SUM_IRMAS ⇒ AVG_AUA_blame	-0.0182	0.03176	-0.08589	0.0428	-0.00451	-0.575	.566
Component	Nationality1 ⇒ SUM_IRMAS	17.0655	2.70975	11.33269	22.9335	0.22991	6.298	<.001
	SUM_IRMAS ⇒ AVG_AUA_blame	0.0127	0.00187	0.00883	0.0164	0.21408	6.783	<.001
	Nationality2 ⇒ SUM_IRMAS	-1.4400	2.49730	-6.41270	3.4381	-0.02105	-0.577	.564
Direct	Nationality1 ⇒ AVG_AUA_blame	0.0903	0.16155	-0.14512	0.3207	0.02056	0.559	.576
	Nationality2 ⇒ AVG_AUA_blame	0.9264	0.14598	0.63048	1.2164	0.22881	6.346	<.001
Total	Nationality1 ⇒ AVG_AUA_blame	0.3066	0.16214	0.06372	0.5468	0.06978	1.891	.059
	Nationality2 ⇒ AVG_AUA_blame	0.9082	0.14943	0.61154	1.2023	0.22431	6.078	<.001

Note. Confidence intervals computed with method: Bootstrap percentiles

Note. Betas are completely standardized effect sizes
