# Supplementary material for: When Appearances Deceive: Rape Myth Schemas Influence Attractiveness Effects Across Cultures
Source: Int J Psychol. 2026 Aug 2;61(5):e70256. doi: 10.1002/ijop.70256 (PMC13429343; doi:10.1002/ijop.70256)
Supplement: Supplementary file 15 — Data S15: Supporting Information 15. [file IJOP-61-e70256-s013.pdf]

GLM Mediation Analysis

Models Info

Mediators Models		
Full Model	m1	SUM_IRMAS ~ Nationality
Indirect Effects	m2	AVG_UAA_blame ~ SUM_IRMAS + Nationality
	IE 1	Nationality ⇒ SUM_IRMAS ⇒ AVG_UAA_blame
Sample size	N	979

Path Model

Statistical Diagram

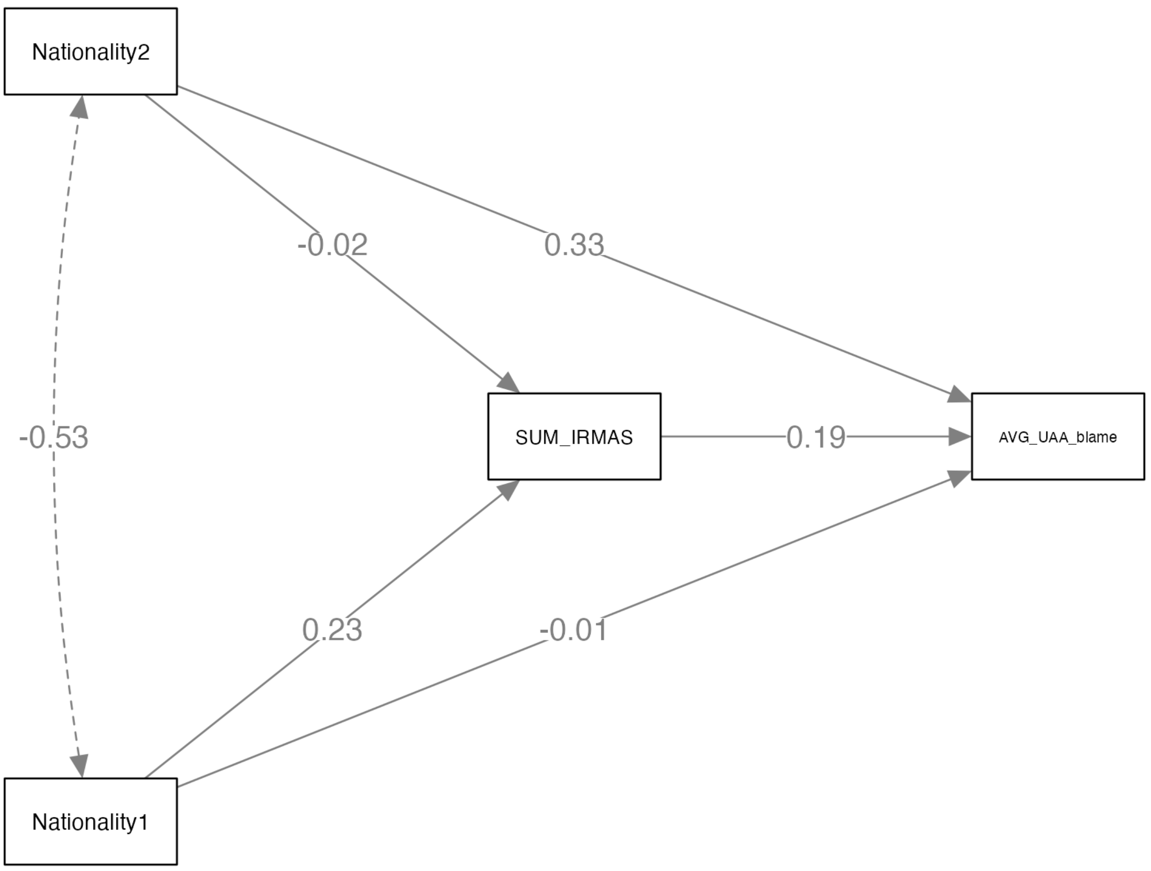

Diagram notes
Categorical independent variables (factors) are represented by contrast indicators
For variable **Nationality** the contrasts are: Nationality1 = HUN - US, Nationality2 = TUR - US

Mediation

Indirect and Total Effects

Type	Effect	Estimate	SE	95% C.I. (a) Lower	Upper	β	z	p
Indirect	Nationality1 ⇒ SUM_IRMAS ⇒ AVG_UAA_blame	0.1936	0.04329	0.10965	0.2899	0.04478	4.474	<.001
	Nationality2 ⇒ SUM_IRMAS ⇒ AVG_UAA_blame	-0.0163	0.02845	-0.07703	0.0371	-0.00410	-0.574	.566
Component	Nationality1 ⇒ SUM_IRMAS	17.0655	2.70975	11.22989	22.8568	0.22991	6.298	<.001
	SUM_IRMAS ⇒ AVG_UAA_blame	0.0113	0.00179	0.00757	0.0151	0.19478	6.356	<.001
	Nationality2 ⇒ SUM_IRMAS	-1.4400	2.49730	-6.41419	3.2625	-0.02105	-0.577	.564
Direct	Nationality1 ⇒ AVG_UAA_blame	-0.0594	0.15441	-0.27985	0.1561	-0.01373	-0.385	.701
	Nationality2 ⇒ AVG_UAA_blame	1.3280	0.13953	1.04875	1.6083	0.33323	9.518	<.001
Total	Nationality1 ⇒ AVG_UAA_blame	0.1343	0.15454	-0.07739	0.3416	0.03105	0.869	.385
	Nationality2 ⇒ AVG_UAA_blame	1.3116	0.14243	1.02786	1.5977	0.32913	9.209	<.001

Note. Confidence intervals computed with method: Bootstrap percentiles

Note. Betas are completely standardized effect sizes
