# Supplementary material for: When Appearances Deceive: Rape Myth Schemas Influence Attractiveness Effects Across Cultures
Source: Int J Psychol. 2026 Aug 2;61(5):e70256. doi: 10.1002/ijop.70256 (PMC13429343; doi:10.1002/ijop.70256)
Supplement: Supplementary file 16 — Data S16: Supporting Information 16. [file IJOP-61-e70256-s009.pdf]

# GLM Mediation Analysis

Models Info

| | | |
|---|---|---|
| Mediators Models | | |
| Full Model | m1 | SUM_IRMAS ~ Nationality |
| Indirect Effects | m2 | AVG_UAUA_blame ~ SUM_IRMAS + Nationality |
| | IE 1 | Nationality ⇒ SUM_IRMAS ⇒ AVG_UAUA_blame |
| Sample size | N | 979 |

## Path Model

**Statistical Diagram**

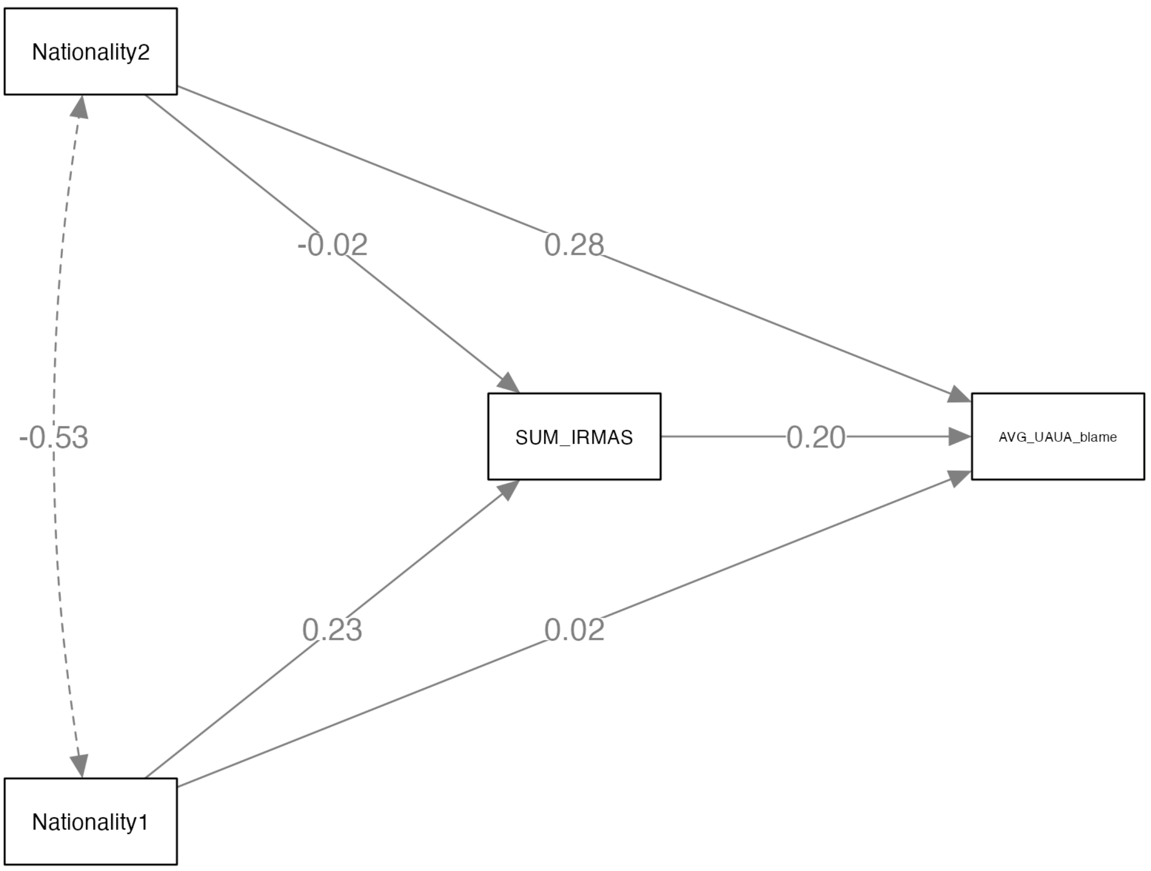


| Diagram notes |
|---|
| Categorical independent variables (factors) are represented by contrast indicators |
| For variable **Nationality** the contrasts are: Nationality1 = HUN - US, Nationality2 = TUR - US |

## Mediation

Indirect and Total Effects

| Type | Effect | Estimate | SE | 95% C.I. (a) Lower | Upper | β | z | p |
|---|---|---|---|---|---|---|---|---|
| Indirect | Nationality1 ⇒ SUM_IRMAS ⇒ AVG_UAUA_blame | 0.1953 | 0.04397 | 0.11568 | 0.2945 | 0.04509 | 4.443 | <.001 |
| | Nationality2 ⇒ SUM_IRMAS ⇒ AVG_UAUA_blame | -0.0165 | 0.02870 | -0.08010 | 0.0389 | -0.00413 | -0.574 | .566 |
| Component | Nationality1 ⇒ SUM_IRMAS | 17.0655 | 2.70975 | 11.39368 | 22.9267 | 0.22991 | 6.298 | <.001 |
| | SUM_IRMAS ⇒ AVG_UAUA_blame | 0.0114 | 0.00183 | 0.00771 | 0.0152 | 0.19610 | 6.268 | <.001 |
| | Nationality2 ⇒ SUM_IRMAS | -1.4400 | 2.49730 | -6.45712 | 3.3681 | -0.02105 | -0.577 | .564 |
| Direct | Nationality1 ⇒ AVG_UAUA_blame | 0.0682 | 0.15793 | -0.15085 | 0.2800 | 0.01574 | 0.432 | .666 |
| | Nationality2 ⇒ AVG_UAUA_blame | 1.1122 | 0.14271 | 0.83081 | 1.4117 | 0.27856 | 7.793 | <.001 |
| Total | Nationality1 ⇒ AVG_UAUA_blame | 0.2635 | 0.15798 | 0.05191 | 0.4702 | 0.06083 | 1.668 | .095 |
| | Nationality2 ⇒ AVG_UAUA_blame | 1.0957 | 0.14560 | 0.80948 | 1.4022 | 0.27443 | 7.526 | <.001 |

*Note.* Confidence intervals computed with method: Bootstrap percentiles

*Note.* Betas are completely standardized effect sizes
